# On the Sequential Hierarchical Cognitive Diagnostic Model

**DOI:** 10.3389/fpsyg.2020.579018

**Published:** 2020-10-07

**Authors:** Xue Zhang, Juntao Wang

**Affiliations:** ^1^China Institute of Rural Education Development, Northeast Normal University, Changchun, China; ^2^School of Mathematics and Statistics, Northeast Normal University, Changchun, China

**Keywords:** sequential hierarchical cognitive diagnostic model, polytomous response data, attribute hierarchy, processing function, model fit

## Abstract

Model data fit plays an important role in any statistical analysis, and the primary goal is to detect the preferred model based on certain criteria. Under the cognitive diagnostic assessment (CDA) framework, a family of sequential cognitive diagnostic models (CDMs) is introduced to handle polytomously scored data, which are attained by answering constructed-response items sequentially. The presence of attribute hierarchies, which can provide useful information about the nature of attributes, will help understand the relation between attributes and response categories. This article introduces the sequential hierarchical CDM (SH-CDM), which adapts the sequential CDM to deal with attribute hierarchy. Furthermore, model fit analysis for SH-CDMs is assessed using eight model fit indices (i.e., three absolute fit indices and five relative fit indices). Two misfit sources were focused; that is, misspecifying attribute structures and misfitting processing functions. The performances of those indices were evaluated *via* Monte Carlo simulation studies and a real data illustration.

## Introduction

Cognitive diagnostic assessment (CDA) has gained widespread use since its introduction, as it can provide fine-grained feedback through pinpointing the presence or absence of multiple fine-grained skills or attributes (Leighton and Gierl, 2007; Templin and Bradshaw, 2013) based on some *cognitive diagnostic models* (CDMs). Many different names according to their different connotations (Rupp et al., 2010; Ma, 2017) can be applied to refer to the CDM, in which the *diagnostic classification model* (DCM; Rupp et al., 2010) is most widely applied.

For dichotomously scored items, a number of CDMs can be found in literature, among others, the deterministic inputs, noisy “and” gate (DINA; Haertel, 1989; Junker and Sijtsma, 2001) model, the deterministic inputs, noisy “or” gate (DINO; Templin and Henson, 2006) model, and the additive CDM (A-CDM; de la Torre, 2011) were most widely used. Furthermore, three most general CDMs, the general diagnostic model (GDM; von Davier, 2008), the log-linear CDM (LCDM; Henson et al., 2009), and the generalized deterministic input noisy and gate model (GDINA; de la Torre, 2011), were proposed to better understand and handle the above models. Specifically, the GDINA model is equivalent to the LCDM when the logit link is used, and the GDM is a general version of both of them.

For polytomously scored items, which yield graded responses with ordered categories or nominal responses, a few models have been developed, such as the GDM for graded response (von Davier, 2008), the nominal response diagnostic model (NRDM; Templin et al., 2008), the partial credit DINA model (de la Torre, 2010), the polytomous LCDM (Hansen, 2013), the sequential CDM (sCDM; Ma and de la Torre, 2016), and the diagnostic tree model (DTM; Ma, 2019). Among them, only sCDM and DTM can model the possible relation between attributes and response categories. Furthermore, Liu and Jiang (2020) proposed the rating scale diagnostic model (RSDM), which was a special version of the NRDM with fewer parameters. Culpepper (2019) presented an exploratory diagnostic framework for ordinal data.

**FIGURE 1:**
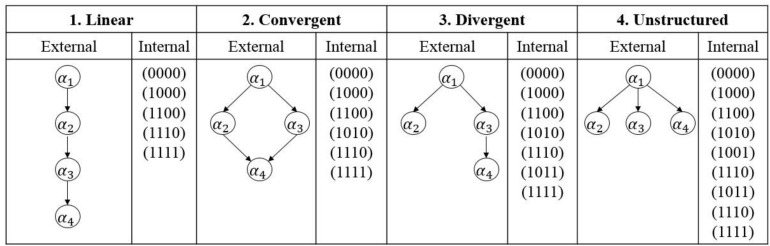
Types of hierarchical attribute structures. For the convergent structure, mastering α_*4*_ needs to master both α_*2*_ and α_*3*_ (Zhan et al., 2020), as well as α_*1*_.

On the other hand, attribute dependencies often occur in practical applications, instead of that all attributes are independent for each examinee. To this end, four different types of attribute hierarchies (i.e., linear, convergent, divergent, and unstructured) were considered to reflect attribute dependencies (Gierl et al., 2007). An example of different types of attribute hierarchies is shown in Figure 1. For an external shape, a directed acyclic graph (DAG) is used to express the attribute hierarchy; and for an internal organization, all possible attribute profiles are provided. Let α_*1*_, α_*2*_, α_*3*_, α_*4*_ denote four attributes measured by a CDA. Take the linear structure as an example, α_*k*_ is the prerequisite of α_*k+1*_ (*k* = 1, 2, 3), as a result, the number of all possible attribute patterns is 5, which is less than 2^4^ = 16. To model attribute hierarchy, Templin and Bradshaw (2014) proposed a hierarchical diagnostic classification model (HDCM). Zhan et al. (2020) proposed a sequential higher-order DINA model with attribute hierarchy to handle the higher-order and hierarchical structures simultaneously using the sequential tree. Interested readers can refer to Rupp et al. (2010) and von Davier and Lee (2019) for detailed information.

It is a central concern to assess global-level fit (i.e., model fit) in the psychometric area. Model fit analysis can be evaluated by two aspects: absolute fit analysis, which assesses how well a given model reproduces the sample data directly; and relative fit analysis, which recommends a better-fit model through comparing at least two candidates. Under the CDA framework, the sources of model misfit include inaccurate item response function (IRF), *Q*-matrix misspecification, misspecifying attribute pattern structures (Han and Johnson, 2019), abnormal response behaviors (e.g., rapid guessing, and cheating), local item dependence, and so on.

Regarding absolute fit assessment, for the sequential GDINA model, Ma and de la Torre (2019) proposed category-level model selection criteria based on the Wald test and the likelihood ratio (LR) test to identify different IRFs; and Ma (2020) used limited-information indices [i.e., *M*_*ord*_ and standardized mean square root of squared residual (SRMSR)] to detect model misspecification or *Q*-matrix misspecification. On the other hand, Lim and Drasgow (2019) proposed the Mantel–Haenszel (MH) chi-square statistic to detect latent attribute misspecification in non-parametric cognitive diagnostic methods.

In terms of relative fit assessment, Sen and Bradshaw (2017) compared the Akaike information criterion (AIC), Bayesian information criterion (BIC), and adjusted BIC (aBIC), and found that they performed poorly to differentiate CDMs and multidimensional item response theory (MIRT) models for dichotomous response data.

Furthermore, some researches focused on the performances of both absolute fit indices (AFIs) and relative fit indices (RFIs) for dichotomous CDMs. Under Bayesian framework, Sinharay and Almond (2007) used Bayesian residuals, which are based on the individuals’ raw scores, as AFI and deviance information criterion (DIC) as RFI to distinguish different measurement models. Chen et al. (2013) used both AFIs [i.e., abs(*fcor*), residuals based on the proportion correct of individual items and the log-odds ratios of item pairs] and RFIs (i.e., -2LL, AIC, and BIC) to identify model and/or *Q*-matrix misspecifications. Hu et al. (2016) investigated the usefulness of AIC, BIC, and consistent AIC (CAIC) as RFIs, and limited information root mean square error of approximation (RMSEA2), abs(*fcor*), and max($\chi_{j\,j^{\prime }}^{2}$) as AFIs to detect model or *Q*-matrix misspecification. Lei and Li (2016) detected model and *Q*-matrix misspecifications using AFIs [i.e., RMSEA, mean of absolute values of Q3 statistic (MADQ3), mean of absolute values of pairwise item covariance residuals (MADres), mean of absolute deviations in observed and expected correlations (MADcor), and mean of all item pair $\chi_{j\,j^{\prime }}^{2}$ statistics (M$\chi_{j\,j^{\prime }}^{2}$)] and RFIs (i.e., AIC and BIC). Empirically, Han and Johnson (2019) assessed global-level fit for dichotomous CDMs using both RFIs (i.e., AIC, BIC, and aBIC) and AFIs [i.e., *M*_2_, RMSEA2, and maximum of all item pair $\chi_{j\,j^{\prime }}^{2}$ statistics (max($\chi_{j\,j^{\prime }}^{2}$))] through a real data illustration.

As no prior studies have analyzed the CDM fit for polytomous response data with attribute hierarchy, the current study concentrates on a novel model, named as the sequential hierarchical CDM (SH-CDM), and assesses model-data fit. This study provides important evidence and insight to support the usefulness of SH-CDMs in the future. The remainder of this article is listed as follows. First, we introduce the sequential hierarchical CDMs and review different model fit indices. Next, simulation studies and a real data illustration are provided to evaluate the performances of those indices. Finally, we end with some concluding remarks. Supplementary Appendix A and Supplementary Material are provided to complement the detailed information and simulation results.

## Materials and Methods

### Model Description

In this section, SH-CDMs are introduced based on Templin and Bradshaw (2014) and Ma and de la Torre (2016). The sCDM (Ma and de la Torre, 2016) is a special version of SH-CDM with non-hierarchical attribute structure, and the HDCM (Templin and Bradshaw, 2014) is a special version of SH-CDM when all response data are scored dichotomously.

To analyze the polytomously scored data from constructed-response items, the sCDM is built upon a sequential process model. As a result, it can provide the detailed problem-solving procedures to support subsequent inference. For a constructed-response test with *J* items, assuming item *j* (*j* = 1, 2, …, *J*) involves *H*_*j*_ tasks that need to be solved sequentially; therefore, if the *1*st task is failed, the score should be 0; if the first *h* (0 < *h* < *H*_*j*_) tasks are successful but the (*h* + *1*)th task is failed, the score should be *h*; else if all tasks are successful, the score should be *H*_*j*_. To this end, Ma and de la Torre (2016) proposed a $\sum_{j=1}^{J}H_{j}$ –by–*K* category-level matrix, named as the *Q*_*c*_-matrix, the element of which is a binary indicator of whether the task of the corresponding item measures this attribute. In this article, only the restricted *Q*_*c*_-matrix is considered. Mathematically, let *Q*_*c*_ = {*q*_*jh,k*_}, *q*_*jh,k*_ = 1 if attribute *k* (*k* = 1, 2, … *K*) is measured by item *j* for task *h* (*h* = 1, 2, …, *H*_*j*_), otherwise, *q*_*jh,k*_ = 0.

Let the processing function, *S*_*jh*_$\left(\vec{{\text{α}}_{g}}\right)$, be the probability of examinees with attribute pattern $\left(\vec{{\text{α}}_{g}}\right)$ answering the first *h* tasks of item *j* correctly given answering the first (*h*-*1*)th tasks correctly, where *g* = 1, 2, …, *G*, and *G* denotes the total number of latent classes. Notating ${\overset{\to *}{\text{α}}}_{l,jh}$ as the reduced attribute profile of examinee *l* (*l* = 1, 2, …, *N*), which contains all the required attributes of item *j* for task *h*. Then, $S_{jh}\left({\vec{\text{α}}}_{g}\right)=S\left({\overset{\to *}{\text{α}}}_{l,jh}\right)$. In this article, the DINA model, DINO model, A-CDM, and GDINA model are considered, which represent the conjunctive, disjunctive, additive, and general condensation rules, respectively. Hereafter, only the identity link is considered for the GDINA model, which is equivalent to the logit link^1^. Assuming that all categories share the same condensation rule, let $K_{j\,h}^{*}$ be the total number of required attributes by item *j* for task *h*; the expressions for processing functions are summarized in Table 1.

**TABLE 1:** Summary of S(${\overset{\to *}{\text{α}}}_{l,jh}$) for different processing functions.

Processing function	Formula	Notations
DINA	$S\left({\overset{\to *}{\text{α}}}_{l,jh}\right)=\phi_{jh,\text{0}}+\phi_{jh,\text{12}...{\text{K}}_{jh}^{*}}\prod_{K=1}^{K_{jh}^{*}}{\text{α}}_{lk}$	**Examinee parameters**: α_*lk*_ is the *k*th attribute of examinee *l*. **Item parameters**: **ϕ**_*jh,0*_ is the intercept; **ϕ**_*jh,k*_ is the main effect of α_*lk*_; **ϕ**_*jh,kk’*_ is the two-way interaction effect of α_*lk*_ and α_*lk’*_; **ϕ**_jh,12…K_jh^*_ is the $K_{j\,h}^{*}$ –way interaction effect of ${\overset{\to *}{\text{α}}}_{l,jh}$
DINO	$S\left({\overset{\to *}{\text{α}}}_{l,jh}\right)\phi_{j\,h,0}+\phi_{j\,h,k}\,\alpha_{l\,k}$	
A-CDM	$S\left({\overset{\to *}{\text{α}}}_{l,jh}\right)\phi_{j\,h,0}+\sum_{k=1}^{K_{j\,h}^{*}}\phi_{j\,h,k}\,\alpha_{l\,k}$	
GDINA	$S\left({\overset{\to *}{\text{α}}}_{l,jh}\right)=\phi_{j\,h,0}+\sum_{k=1}^{K_{j\,h}^{*}}\phi_{j\,h,k}\,\alpha_{l\,k}+\sum_{k^{\prime }=k+1}^{K_{j\,h}^{*}}\sum_{k=1}^{K_{j\,h}^{*}-1}\phi_{j\,h,k\,k^{\prime }}\,\alpha_{l\,k}\,\alpha_{l\,k^{\prime }}+\ldots +\phi_{j\,h,12\,\ldots \,K_{j\,h}^{*}}\,\prod_{k=1}^{K_{j\,h}^{*}}\alpha_{l\,k}$	

Furthermore, the existence of attribute hierarchy will reduce the model complexity of IRF. To deal with attribute hierarchies, the HDCM was proposed by Templin and Bradshaw (2014). As the HDCM is nested within more general CDMs, the SH-CDM is introduced by combining it with the sCDM. In the SH-CDM, the influence of attribute hierarchy is reflected in the processing function. Take the SH-GDINA model as an example, assuming four linear attributes (Figure 1) are measured in the test and three attributes are required by item *j* for task *h*, $S\left({\overset{\to *}{\text{α}}}_{l,jh}\right)=\phi_{j\,h,0}+\phi_{j\,h,k_{1}}\,\alpha_{l\,k_{1}}+\phi_{j\,h,k_{1}\,k_{2}}\,\alpha_{l\,k_{1}}\,\alpha_{l\,k_{2}}+\phi_{j\,h,k_{1}\,k_{2}\,k_{3}}\,\alpha_{l\,k_{1}}\,\alpha_{l\,k_{2}}\,\alpha_{l\,k_{3}}$, where the subscript of *k* denotes the order of the required attributes.

To ensure joint identifiability of the HDCMs, Gu and Xu (2019) restricted that the sparsified version of *Q*-matrix had at least three entries of “1”s in each column and *Q*-matrix can be rearranged as $Q=\left(\frac{Q^{0}}{Q^{*}}\right)$, where *Q*^0^ was equivalent to a *K*-by-*K* identity matrix **I***_*K*_* under the attribute hierarchy and the densified version of *Q*^∗^ contained *K* distinct column vectors. In addition, if *Q*-matrix was constrained to contain an **I***_*K*_*, the HDCMs were identified. The SH-CDM identifiability shares the same restrictions as those mentioned above. Interested reader can refer to Gu and Xu (2019) for further discussion on model identifiability.

### Model Fit Indices

To assess global-level fit of CDMs, both absolute fit assessment and relative fit assessment are done to identify adequate-fit models and select the best-fit model, respectively. To ensure the comparability of different AFIs, SRMSR, 100^∗^MADRESIDCOV, and MADcor are considered as they share the same rule. To choose the best-fit model among all candidates, five widely used RFIs [i.e., AIC, BIC, the second-order information criterion (AICc), aBIC, and CAIC] are evaluated and compared.

#### Absolute Fit Index

The SRMSR (Maydeu-Olivares, 2013) is a measure of pairwise correlations. As a standardized statistic, test length has few influences on the performance of SRMSR. The SRMSR can be calculated as

(1) $$\text{SRMSR}=\sqrt{\frac{2}{J\,\left(J-1\right)}\,\sum_{j<j^{\prime }}{\left(r_{j\,j^{\prime }}-{\hat{r}}_{j\,j^{\prime }}\right)}^{2}},$$

where *r*_*jj’*_ and ${\hat{r}}_{j\,j^{\prime }}$ denote the observed and predicted pairwise item correlations, respectively. The model with smaller SRMSR will be identified as a good fit one.

The mean absolute deviation (MAD) is a fundamental statistic to calculate the last two AFIs mentioned before, which measures the discrepancy between the observed item conditional probabilities of success and the predicted ones. The RESIDCOV denotes the residual covariance of pairwise items. Then, we can obtain the mean of absolute deviations of residual covariances (MADRESIDCOV; McDonald and Mok, 1995) by replacing the conditional probabilities in MAD by the RESIDCOVs. The MADRESIDCOV measures the discrepancy between observed and predicted pairwise item residual covariance (RESIDCOV). Let,

(2) $$\gamma_{j\,j^{\prime }}≜{\text{RESIDCOV}}_{j\,j^{\prime }}=\frac{n_{j\,j^{\prime },11}\,n_{j\,j^{\prime },00}-n_{j\,j^{\prime },10}\,n_{j\,j^{\prime },01}}{n^{2}}-\frac{e_{j\,j^{\prime },11}\,e_{j\,j^{\prime },00}-e_{j\,j^{\prime },10}\,e_{j\,j^{\prime },01}}{n^{2}},$$

Then, we can obtain

(3) $$\text{MADRESIDCOV}=\frac{\sum_{j=1}^{J}\sum_{j\neq j^{\prime }}\left|\gamma_{j\,j^{\prime }}-{\hat{\gamma }}_{j\,j^{\prime }}\right|}{J\,\left(J-1\right)}.$$

100^∗^MADRESIDCOV is used equivalently, as the magnitude of MADRESIDCOV is usually small. The MADcor (DiBello et al., 2007) is the mean of absolute deviations in observed and expected correlations of pairwise items. The MADcor equals$\frac{2}{J\,\left(J-1\right)}\,\sum_{j<j^{\prime }}\left|r_{j\,j^{\prime }}-{\hat{r}}_{j\,j^{\prime }}\right|$, where *r*_*jj’*_ and ${\hat{r}}_{j\,j^{\prime }}$ have the same meanings as those in SRMSR. For MAD-type indices, a smaller value (i.e., value near to zero) denotes better fit.

#### Relative Fix Index

Different types of information criteria are calculated with respect to the penalty term, the expressions of which are presented in Table 2. AIC (Akaike, 1974) and BIC (Schwarz, 1978) are most widely applied since their introductions. The second-order information criterion (AICc; Sugiura, 1978) was derived to deal with the small ratio of sample size to estimated number of parameters case (i.e., less than 40; Burnham and Anderson, 2002). As the sample size gets large, AICc converges to AIC. The aBIC (Sclove, 1987) modified BIC by adjusting the sample size term to handle the small sample size case well. The CAIC was proposed by Bozdogan (1987), in which then penalty terms include both the order of the model and the sample size. The candidate model with smaller RFI is recommended.

**TABLE 2:** Formulas of different relative fit indices.

Index	Formula	Notation
AIC	AIC = –2ln(*LL*) + 2*P*	
BIC	BIC = –2ln(*LL*) + *P* ln(*N*)	*LL*: likelihood
AICc	AICc = AIC + [2*P*(*P* + 1)]/(*N* – *P* – 1)	*P*: the effective
aBIC	aBIC = –2ln(*LL*) + *P* ln[(*N* + 2)/24]	number of parameters
CAIC	CAIC = –2ln(*LL*) + (ln(*N*) + 1)*P* = BIC – *P*	*N*: sample size

*AIC, Akaike information criterion; BIC, Bayesian information criterion; aBIC, adjusted BIC; CAIC, consistent AIC.*

### Simulation Studies

The simulation studies aim (a) to examine parameter recovery for SH-CDMs and (b) to compare the performances of different AFIs and RFIs for SH-CDMs. Two different sources of misfit are considered: the first type of misfit is due to attribute structures misspecification, and the second type of misfit relies on different processing functions. To this end, three simulation studies are conducted: (I) to examine whether parameters of SH-CDMs can be recovered well; (II) to investigate the performances of model fit indices to identity attribute structures; and (III) to investigate their performances to detect the processing function misspecification, respectively.

The simulation conditions are summarized in Table 3. More details will be given in the simulation design sections. In this article, the GDINA R package (Ma and de la Torre, 2020) was used to estimate different SH-CDMs and assess the model fit. The source code including the computation of indices, which were not provided in the GDINA R package, was provided in Supplementary Appendix A. The mapping matrix method (Tutz, 1997) and the expected *a posteriori* (EAP) method, which are the default methods in the GDINA package for sequential CDMs, were used to estimate item parameters and attribute profiles, respectively. Five hundred replications were conducted for each condition.

**TABLE 3:** Summary of simulation conditions.

Factors	Studies I and II	Study III
Sample size (*N*)	1,000	1,000; 3,000
Attribute structure	Non-hierarchical; linear; convergent; divergent; unstructured	
Generation processing function	GDINA	DINA, DINO, A-CDM, GDINA
Calibration processing function	GDINA	DINA, DINO, A-CDM, GDINA
Item quality	High: *U*(0.1, 0.2), low: *U*(0.2, 0.3)	
Attribute generation	Uniform structure	
Test length (*J*)	24	
Number of attributes (*K*)	4	
Number of categories (*G*)	4	

### Study Design I

In this study, the GDINA model was chosen as the processing function, as its generality. Attribute profiles were generated from the uniform structure; that is, all the possible latent classes shared the same probability. Sample size was 1,000. A 24-item test, in which there were 20 four-category items and four dichotomously scored items, was used. For four-category items, four attributes were measured totally and no more than three attributes were required by each item. Without loss of generality, the last four items were dichotomously scored with an identity sub*Q*-matrix to ensure model identifiability. Two manipulated factors included attribute structure (non-hierarchical, linear, convergent, divergent, and unstructured) and item quality (high and low). For each data set, item parameters and attribute profiles were simulated separately and the *Q*-matrix was kept consistent.

The item parameter recovery was calculated in terms of average root mean square error (RMSE), average bias, and average relative absolute bias (RAbias), and the classification accuracies were examined using pattern-wise agreement rate (PAR) and attribute-wise agreement rate (AAR).

### Simulation Result I

Hereafter, for convenience, let S1 = the SH-GDINA model with non-hierarchical attribute structure; S2 = the SH-GDINA model with linear attribute structure; S3 = the SH-GDINA model with convergent attribute structure; S4 = the SH-GDINA model with divergent attribute structure; S5 = the SH-GDINA model with unstructured attribute structure.

Table 4 summarizes the estimation accuracy and precision of SH-GDINA models. For different attribute structures, attribute profiles in high item quality cases could be recovered better than in the corresponding low item quality cases. The estimation accuracy of item parameters had a similar trend except for convergent attribute structure cases, although the values of RMSE and bias in low item quality cases were smaller than those in high item quality cases, which is because true item parameters’ values in the low cases were smaller, and the smaller true values led to larger RAbiases.

**TABLE 4:** Summary of parameter recovery when CM = GM.

Model	Low item quality					High item quality				
		
	AAR	PAR	RMSE	bias	RAbias	AAR	PAR	RMSE	bias	RAbias
S1	0.9008	0.6742	0.1683	0.0000	6.8400	0.9787	0.9226	0.1336	0.0001	2.0298
S2	0.9623	0.8593	0.1842	0.0688	4.6691	0.9915	0.9664	0.2448	0.0961	4.5692
S3	0.9514	0.8240	0.1998	0.0655	4.4051	0.9904	0.9626	0.2552	0.0906	12.9081
S4	0.9518	0.8248	0.1715	0.0390	6.1866	0.9899	0.9604	0.2176	0.0544	3.7261
S5	0.9422	0.7938	0.1801	0.0396	10.9168	0.9903	0.9624	0.2242	0.0556	6.3453

*AIC, Akaike information criterion; BIC, Bayesian information criterion; aBIC, adjusted BIC; CAIC, consistent AIC.*

Furthermore, the SH-GDINA model with non-hierarchical attribute structure, which was the most general one among these models, was used to fit response data generated by different SH-GDINA models. The parameter recovery is summarized in Table 5. Compared with results shown in Table 4, AAR and PAR were smaller, and RMSE and RAbias were larger. It appears that specifying the attribute hierarchy can significantly improve the estimation accuracy and precision, which supports the introduction of SH-CDMs.

**TABLE 5:** Summary of parameter recovery when data were fitted by S1.

GM	Low item quality					High item quality				
		
	AAR	PAR	RMSE	bias	RAbias	AAR	PAR	RMSE	bias	RAbias
S2	0.9475	0.8154	0.4556	−0.0004	13.6676	0.9862	0.9486	0.4808	−0.0001	8.2845
S3	0.9490	0.8169	0.4077	0.0000	9.6069	0.9894	0.9595	0.4383	−0.0002	9.2714
S4	0.9369	0.7796	0.4300	−0.0002	11.2261	0.9837	0.9385	0.4452	0.0000	30.6335
S5	0.9255	0.7426	0.4159	−0.0001	13.1993	0.9825	0.9343	0.4057	0.0001	6.0283

*GM, data generation model; AAR, attribute-wise agreement rate; PAR, pattern-wise agreement rate; RMSE, root mean square error; RAbias, average relative absolute bias.*

### Study Design II

In this study, the same simulation settings as the simulation study I were considered. Correct detection rates (CDRs) were used to evaluate the performances of different indices. For AFIs, as there is no sufficient evidence for the cutoff values of these AFIs to support model fit assessment, the CDR is calculated as the rate of the smallest values for all replications. Meanwhile, the box plot of AFIs is also provided to compare the performances of different AFIs intuitively.

### Simulation Result II

A popular rule for AIC (Burnham and Anderson, 2002) is that a difference of 2 or less is considered negligible and a difference exceeding 10 constitutes strong support. In this article, the same rule is used for all the RFIs. For the non-hierarchical attribute structure, all AFIs of the data generation model had the smallest values, and all RFIs recommended the true model with strong support. For hierarchical attribute structures, Tables 6, 7 provide CDRs of RFIs and AFIs, respectively.

**TABLE 6:** Correct detection rates of RFIs for different hierarchical attribute structures.

GM	CM	Low item quality					High item quality				
			
		AIC	BIC	aBIC	CAIC	AICc	AIC	BIC	aBIC	CAIC	AICc
S2	S1	0	0	0	0	0	0	0	0	0	0
	S2	**0.99**	**1**	**1**	**1**	**1**	**0.998**	**1**	**1**	**1**	**1**
	S3	0.01	0	0	0	0	0.008	0	0	0	0
	S4	0	0	0	0	0	0	0	0	0	0
	S5	0	0	0	0	0	0	0	0	0	0
S3	S1	0	0	0	0	0	0	0	0	0	0
	S2	0	0	0	0	0	0	0	0	0	0
	S3	**0.99**	**1**	**1**	**1**	**1**	**1**	**1**	**1**	**1**	**1**
	S4	0.01	0	0	0	0	0	0	0	0	0
	S5	0	0	0	0	0	0	0	0	0	0
S4	S1	0	0	0	0	0	0	0	0	0	0
	S2	0	0	0	0	0	0	0	0	0	0
	S3	0	0	0	0	0	0	0	0	0	0
	S4	**1**	**1**	**1**	**1**	**1**	**1**	**1**	**1**	**1**	**1**
	S5	0	0	0	0	0	0	0	0	0	0
S5	S1	0	0	0	0	0	0	0	0	0	0
	S2	0	0	0	0	0	0	0	0	0	0
	S3	0	0	0	0	0	0	0	0	0	0
	S4	0	0	0	0	0	0	0	0	0	0
	S5	**1**	**1**	**1**	**1**	**1**	**1**	**1**	**1**	**1**	**1**

*GM, data generation model; CM, calibration model; RFI, relative fit index; AIC, Akaike information criterion; BIC, Bayesian information criterion; aBIC, adjusted BIC; CAIC, consistent AIC. The bold value means the largest value in a specific condition.*

**TABLE 7:** CDRs of AFIs for different hierarchical attribute structures.

GM	CM	Low item quality			High item quality		
			
		MADcor	100MAD RESIDCOV	SRMSR	MADcor	100MAD RESIDCOV	SRMSR
S2	S1	**0.282**	0.226	**0.270**	**0.292**	0.210	0.304
	S2	0.206	**0.238**	0.228	0.248	**0.306**	**0.224**
	S3	0.150	0.162	0.174	0.128	0.150	0.136
	S4	0.178	0.164	0.150	0.134	0.128	0.124
	S5	0.184	0.210	0.178	0.198	0.206	0.192
S3	S1	**0.296**	0.260	**0.308**	**0.302**	0.266	**0.298**
	S2	0.006	0.008	0	0	0	0
	S3	0.280	**0.312**	0.274	0.294	**0.314**	0.292
	S4	0.196	0.226	0.230	0.212	0.200	0.224
	S5	0.222	0.194	0.188	0.192	0.220	0.186
S4	S1	**0.426**	**0.364**	**0.396**	**0.410**	0.37	**0.420**
	S2	0	0	0	0	0	0
	S3	0	0	0	0	0	0
	S4	0.314	0.360	0.326	0.306	**0.33**	0.296
	S5	0.260	0.276	0.278	0.284	0.30	0.284
S5	S1	**0.510**	0.446	0.496	0.454	0.42	0.458
	S2	0	0	0	0	0	0
	S3	0	0	0	0	0	0
	S4	0.002	0.014	0.004	0	0	0
	S5	0.488	**0.540**	**0.500**	**0.546**	**0.58**	**0.542**

*GM, data generation model; CM, calibration model; CDR, correct detection rate; AFI, absolute fit index; SRMSR, standardized mean square root of squared residual. The bold value means the largest value in a specific condition.*

As shown in Table 6, all RFIs could select the true model with a probability larger than 0.99. Regarding the effectiveness of detecting distinguished models with similar RFIs, we calculated the rates of the differences between RFI values of two candidates, which were smaller than 10, and named as *the indistinguishable proportion*. When response data were generated by S2, the indistinguishable proportions of AIC to differentiate S3 were 4.8% and 27.2% for the high item quality case and the low item quality case, respectively; and the indistinguishable proportion of AICc was 0.2% for the high item quality case. To generate response data using S3, for the high item quality case, the indistinguishable proportion of AIC to differentiate S4 was 3.8%; for the low item quality case, the indistinguishable proportion of AIC to differentiate S4 was 29%, among them 1% of the time AIC could not differentiate S3, S4, and S5. For the case in which data were generated by S4, the indistinguishable proportion of AIC to differentiate S4 and S5 was less than 6%. Other cases could be differentiated well.

**FIGURE 2:**
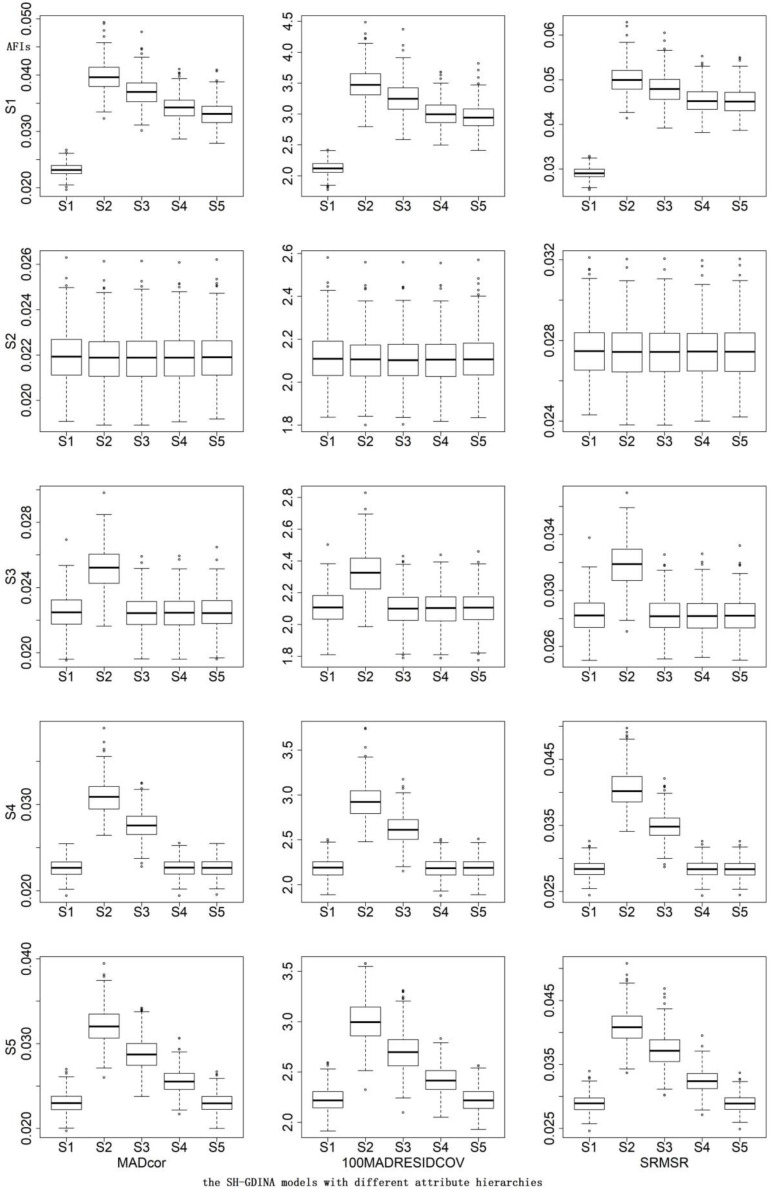
Box plots of absolute fit indices (AFIs) in low item quality cases of simulation study II.

**FIGURE 3:**
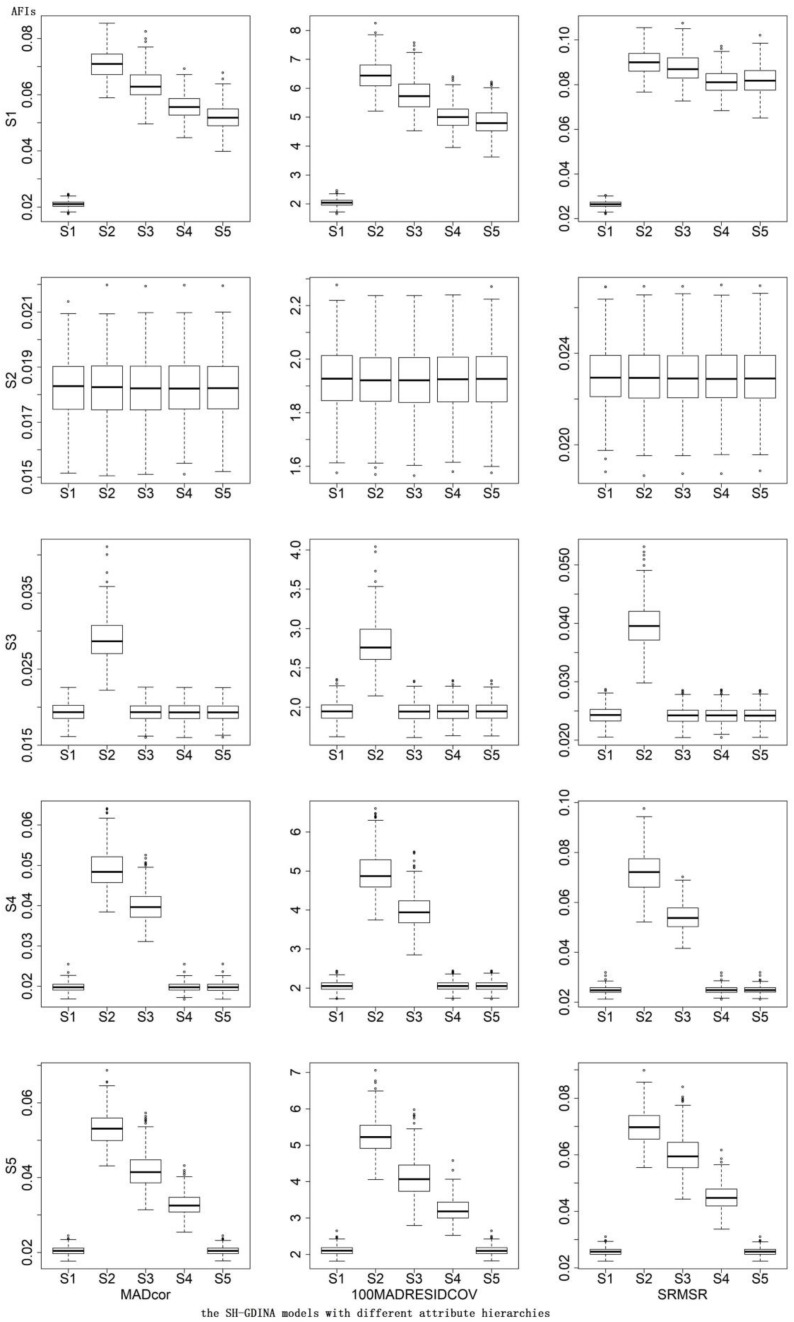
Box plots of absolute fit indices (AFIs) in high item quality cases of simulation study II.

In terms of AFIs (Table 7), S1 mostly had the smallest AFIs. The CDRs of different AFIs were similar. According to the box plots of AFIs (Figures 2, 3), when generating data using S2, it was very hard to differentiate these models. Similarly, it was difficult to distinguish S3 from S1 and S5, S4 from S1 and S5, or S5 from S1. High item quality led to large values of AFIs. It appears that RFIs outperform AFIs for SH-GDINA models, and RFIs distinguish SH-GDINA models with convergent attribute structures from models with unstructured attribute structures with difficulty.

### Simulation Design III

In this study, attribute generation method, test length, and test structure were kept the same as those in simulation studies I and II. The manipulated factors included sample size (1,000 and 3,000), attribute structure (non-hierarchical, linear, convergent, divergent, and unstructured), item quality (high and low), and data generation/calibration model (SH-DINA, SH-DINO, SH-ACDM, and SH-GDINA). To evaluate the performances of different indices, the CDRs were reported. Hence, item parameters and the *Q*-matrix were fixed for each condition, and attribute profiles were simulated separately.

### Simulation Result III

For convenience, let M1 = the SH-DINA model, M2 = the SH-DINO model, M3 = the SH-ACDM, and M4 = the SH-GDINA model.

#### Relative Fit Index

Limited by the space, we only provided the results when the probabilities of true model selection by different RFIs were different or incorrect in Table 8. The whole results (i.e., CDRs of both RFIs and AFIs, box plots of AFIs) were provided in the Supplementary Material. The different performances were reflected in the SH-ACDM and the SH-GDINA model. Under the conditions mentioned in Table 8, AIC and AICc always chose the true model, BIC and CAIC always chose the alternative model, and aBIC mostly selected the true model. Besides, for linear attribute structures, when the data generation model was the SH-ACDM, all RFIs recommended the SH-GDINA model with a probability larger than 0.98. In other cases, all RFIs could select the data generation model as the better fitting one with a probability larger than 0.93.

**TABLE 8:** Selected results of CDRs of RFIs for different models.

Condition	CM	AIC	BIC	aBIC	CAIC	AICc
*Non-hierarchical* structure Low item quality, *N* = 1,000 GM = M4	M1	0	0	0	0	0
	M2	0	0	0	0	0
	M3	0	**1**	**0.502**	**1**	0.134
	M4	**1**	0	0.498	0	**0.866**
*Non-hierarchical* structure Low item quality, *N* = 3,000 GM = M4	M1	0	0	0	0	0
	M2	0	0	0	0	0
	M3	0	**0.76**	0	**0.978**	0
	M4	**1**	0.24	**1**	0.022	**1**
*Convergent* structure Low item quality, *N* = 1,000 GM = M4	M1	0	0	0	0	0
	M2	0	0	0	0	0
	M3	0	**0.642**	0.05	**0.822**	0.014
	M4	**1**	0.358	**0.95**	0.178	**0.986**
*Divergent* structure Low item quality, *N* = 1,000 GM = M4	M1	0	0	0	0	0
	M2	0	0	0	0	0
	M3	0	**0.888**	0.02	**0.988**	0
	M4	**1**	0.112	**0.98**	0.012	**1**
*Unstructured* structure Low item quality, *N* = 1,000 GM = M4	M1	0	0	0	0	0
	M2	0	0	0	0	0
	M3	0	**0.894**	0.002	**0.998**	0
	M4	**1**	0.106	**0.998**	0.002	**1**
*Unstructured* structure High item quality, *N* = 3,000 GM = M4	M1	0	0	0	0	0
	M2	0	0	0	0	0
	M3	0	**0.652**	0.002	**0.938**	0
	M4	**1**	0.348	**0.998**	0.062	**1**

*GM, data generation model; CM, calibration model; CDR, correct detection rate; RFI, relative fit index; AIC, Akaike information criterion; BIC, Bayesian information criterion; aBIC, adjusted BIC; CAIC, consistent AIC. The bold value means the largest value in a specific condition.*

When data were generated by M4 with non-hierarchical attribute structures, in low item quality cases, the indistinguishable proportions of aBIC and AICc to differentiate M3 and M4 were about 10% for a small sample size; for a large sample size, BIC could not differentiate M3 and M4 5.4% of the time and the indistinguishable proportion of CAIC was 1%.

For linear attribute structures, AIC could not distinguish M1 from M3 or M4 for one or two replications under different conditions. When the generation model was M2, the indistinguishable proportions of AIC with high item quality were similar to those in the “GM = M1” case. When response data were generated by M3 or M4, all RFIs could not differentiate M3 and M4.

For convergent attribute structures, AIC could not distinguish M2 from M4 in no more than one replication under different conditions. M3 and M4 could not be distinguished well. When generating response data using M3, the indistinguishable proportions of AIC to differentiate M4 ranged from 45% to 69%, the corresponding indistinguishable proportions of AICc ranged from 3% to 27%, and BIC was not able to differentiate M3 from M4 11% of the time for the high item quality case with small sample size. When “GM = M4,” for the low item quality case with the large sample size, aBIC was not able to differentiate M4 and M3 32% of the time, and the indistinguishable proportion of CAIC was 8%; the indistinguishable proportions of AIC, BIC, aBIC, CAIC, and AICc for the low item quality case with the small sample size were about 1.6%, 39%, 7.8%, 30.2%, and 7.6%, respectively; and for the high item quality case with the small sample size, the indistinguishable proportions of BIC and aBIC were 11.4% and 24.8%, respectively.

For divergent attribute structures, in one replication, AIC could not distinguish M1 from M4. Furthermore, AIC was not able to distinguish M3 from M4 about 8.4% of the time, and the corresponding indistinguishable proportion of AICc was 2%. When data were generated by M4, the indistinguishable proportions of BIC and CAIC for high item quality with the small sample size were 7.2% and 14%; for low item quality, the corresponding proportions of BIC, aBIC, CAIC, and AICc with the small sample size were 7.6%, 5.2%, 2.8%, and 1.4%, respectively, and those of BIC and CAIC with the large sample size were 2.2% and 5.6%, respectively.

For unstructured attribute structures, distinguishing M3 from M4 using AIC would fail about 1.4% of the time, and the indistinguishable proportions of BIC, aBIC, and CAIC to differentiate M4 from M3 were about 11%, 2%, and 3%, respectively. Overall, AICc can select the true model and almost distinguish it from others for all manipulated conditions.

#### Absolute Fit Index

As M4 almost had the smallest values of AFIs for all conditions except the linear attribute structure cases, only the results of these cases were presented in Table 9. Both small sample size and high item quality led to larger values of AFIs. For linear attribute structures, it was hard to differentiate M3 and M4, when to generate data using M4, the difference between AFIs of M3 and M4 decreased as item quality became low. For other structures, the trends were similar. This observation indicates that AFIs cannot easily differentiate SH-CDMs that differ by processing functions.

**TABLE 9:** CDRs of AFIs for different models with *linear* attribute structures.

GM	CM	Low item quality			High item quality		
			
		MADcor	100MAD RESIDCOV	SRMSR	MADcor	100MAD RESIDCOV	SRMSR
				*N* = 1,000			
M1	M1	0.158	0.142	0.114	0.176	0.144	0.126
	M2	0	0	0	0	0	0
	M3	**0.53**	**0.526**	**0.528**	**0.42**	**0.472**	**0.454**
	M4	0.312	0.332	0.358	0.404	0.384	0.42
M2	M1	0	0	0	0	0	0
	M2	0.082	0.082	0.056	0.042	0.044	0.036
	M3	0.444	0.438	0.454	**0.536**	**0.522**	**0.514**
	M4	**0.474**	**0.48**	**0.49**	0.422	0.434	0.45
M3	M1	0	0	0	0	0	0
	M2	0	0	0	0	0	0
	M3	**0.554**	**0.548**	**0.558**	**0.554**	**0.592**	**0.54**
	M4	0.446	0.452	0.442	0.446	0.408	0.46
M4	M1	0	0	0	0	0	0
	M2	0	0	0	0	0	0
	M3	0.46	0.492	0.492	**0.562**	**0.568**	**0.556**
	M4	**0.54**	**0.508**	**0.508**	0.438	0.432	0.444
				*N* = 3,000			
M1	M1	0.146	0.158	0.114	0.154	0.162	0.128
	M2	0	0	0	0	0	0
	M3	**0.438**	**0.44**	**0.488**	**0.464**	**0.44**	**0.508**
	M4	0.416	0.402	0.398	0.382	0.398	0.364
M2	M1	0	0	0	0	0	0
	M2	0.078	0.072	0.048	0.078	0.072	0.048
	M3	0.422	0.438	0.46	0.422	0.438	0.46
	M4	**0.5**	**0.49**	**0.492**	**0.5**	**0.49**	**0.492**
M3	M1	0	0	0	0	0	0
	M2	0	0	0	0	0	0
	M3	0.492	0.462	0.48	**0.502**	0.498	**0.53**
	M4	**0.508**	**0.538**	**0.52**	0.498	**0.502**	0.47
M4	M1	0	0	0	0	0	0
	M2	0	0	0	0	0	0
	M3	**0.558**	0.49	**0.554**	**0.562**	**0.542**	**0.544**
	M4	0.442	**0.51**	0.446	0.438	0.458	0.456

*GM, data generation model; CM, calibration model; CDR, correct detection rate; AFI, absolute fit index; SRMSR, standardized mean square root of squared residual. The bold value means the largest value in a specific condition.*

### Real Data Illustration

#### Data Source

This example application is from the TIMSS 2007 eighth-grade mathematics assessment, which is from Booklet 1 that measured three attributes (Lee et al., 2013): Attribute 1 (α_*1*_): whole numbers and integers; Attribute 2 (α_*2*_): fractions, decimals, and percentages; and Attribute 3 (α_*3*_): data analysis and probability. There are 12 dichotomously scored items and one three-category item answered by 544 students from the United States.

**FIGURE 4:**
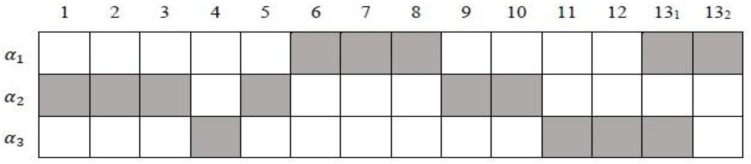
The *Q*_*c*_-matrix for Booklet 1 data from TIMSS 2007. 13_1_ and 13_2_ denote the *1*st and *2*nd tasks of item 13, respectively.

The *Q*_*c*_-matrix based on the works by Lee et al. (2013) and Ma (2019) is presented in Figure 4. According to general knowledge about numeric, α_*2*_ cannot be the prerequisite of α_*1*_. Hence, including the non-hierarchical attribute structure (#structure = 1), 11 different attribute structures are analyzed. The detailed DAGs of different hierarchical attribute structures are shown in Table 10. Without loss of generality, the GDINA model is chosen as the processing function in this section because, in simulation studies, we noticed that it is not easy to differentiate the SH-GDINA model from others. This dataset is analyzed using the GDINA R package (Ma and de la Torre, 2020).

**TABLE 10:** Summary of hierarchical attribute structures.

#structure	DAG	#structure	DAG
2	α_1_→α_2_→α_3_	7	α_2_→α_3_,α_1_
3	α_1_→α_3_→α_2_	8	α_3_→α_2_,α_1_
4	α_3_→α_1_→α_2_	9	α_3_→α_1_,α_2_
5	α_1_→α_2_,α_3_	10	α_1_→α_2_,α_1_→α_3_
6	α_1_→α_3_,α_2_	11	α_3_→α_1_,α_3_→α_2_

*DAG, directed acyclic graph.*

## Results

Table 11 shows the comparison among different structures using both AFIs and RFIs. The smallest values are in boldface. Different indices performed similarly to assess model-data fit that is because most of the items required only one attribute. From an item-level perspective, if only one attribute is required by one item, there is no difference among candidate models with different attribute structures.

**TABLE 11:** Model fit indices of SH-GDINA models for TIMSS data.

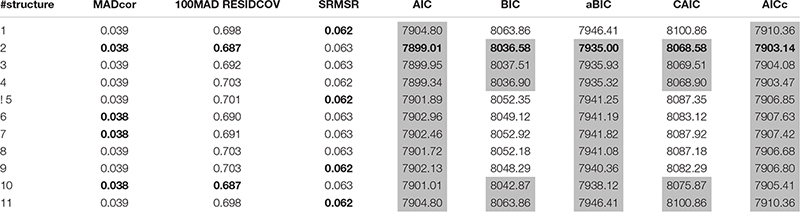

*The recommended attribute structures by RFIs were highlighted in gray. SRMSR, standardized mean square root of squared residual; AIC, Akaike information criterion; BIC, Bayesian information criterion; aBIC, adjusted BIC; CAIC, consistent AIC; AICc, second-order information criterion. The bold value means the smallest value of an index.*

On the other hand, Wang and Lu (2020) proposed two predetermined cutoff values (i.e., 0.025 and 0.05) of estimated proportions of latent classes to identify the labels for estimated latent classes. The estimated proportions of latent classes were shown in Table 12. All estimated proportions of (000), (100), (001), and (111) were larger than 0.025, and except (000) and (111), only (001) modeled using *4*th structure had an estimated proportion larger than 0.05. It is not enough to distinguish different structures, which may be because the sample size of this dataset was small.

**TABLE 12:** Summary of estimated proportion of each latent class.

#structure	000	100	010	001	110	101	011	111
1	0.487	0.030	0.001	0.035	0.014	0.000	0.021	0.412
2	0.535	0.033	–	–	0.016	–	–	0.417
3	0.535	0.041	–	–	–	0.000	–	0.424
4	0.517	–	–	0.052	–	0.000	–	0.431
5	0.489	0.034	–	0.047	0.012	0.000	–	0.418
6	0.531	0.032	0.007	–	0.016	0.000	–	0.415
7	0.514	0.028	0.004	–	0.017	–	0.029	0.408
8	0.483	0.038	–	0.042	–	0.000	0.019	0.419
9	0.510	–	0.003	0.039	–	0.000	0.025	0.423
10	0.535	0.033	–	–	0.016	0.000	–	0.417
11	0.511	–	–	0.040	–	0.000	0.025	0.424

*“–” denotes the latent class is impermissible.*

## Conclusion

In order to avoid possible misleading conclusion, model-data fit must be thoroughly assessed before drawing the model-based inference. Although there are abundant research examining model fit assessment for CDMs, there is a lack of an effective guidance on how to deal with polytomously scored items with hierarchical attribute structures, and the aim of the present study is to fill in this gap. In this paper, we developed a sequential hierarchical cognitive diagnostic model to handle polytomous response data with hierarchical attribute structures and further evaluated model-data fit using both absolute fit indices and relative fit indices.

Across all simulation conditions, the SH-CDM can be recovered well, and aBIC and AICc are recommended for the SH-CDMs due to their high CDRs and acceptable distinguishable proportions. To distinguish different attribute structures for SH-GDINA models, RFIs outperform AFIs. Furthermore, AFIs used in this study are inappropriate to differentiate processing functions of the SH-CDM.

This study was the first attempt at assessing global-level fit of hierarchical CDMs and polytomous response data. However, the results are limited to SH-CDMs using the same condensation rules; future research pertaining to mixture measurement model and different condensation rules for different tasks in one item should be expanded to enhance the practicability of SH-CDMs. Also, it is necessary to extend the study to deal with sparse *Q*-matrix with large *K*. In addition, local-level (i.e., item-level) fit should be further examined to complement global fit analysis. On the other hand, as smaller values (close to zero) of AFIs indicate a good model-data fit, it would be worthwhile to identify the corresponding cutoff values using the resampling technique.

## Data Availability Statement

Publicly available datasets were analyzed in this study. This data can be found here: https://timss.bc.edu/TIMSS2007/idb_ug.html.

## Author Contributions

XZ provided original thoughts and completed the writing of this article. JW provided key technical support. Both authors contributed to the article and approved the submitted version.

## Conflict of Interest

The authors declare that the research was conducted in the absence of any commercial or financial relationships that could be construed as a potential conflict of interest.

## Footnotes

1 A simulation check has been done to test the equivalence using the GDINA package; results indicated there was no significant difference between the identity link and the logit link.
