# Supplementary material for: On the Sequential Hierarchical Cognitive Diagnostic Model
Source: Front Psychol. 2020 Oct 7;11:579018. doi: 10.3389/fpsyg.2020.579018 (PMC7577049; doi:10.3389/fpsyg.2020.579018)
Supplement: Supplementary file 1 [file Data_Sheet_1.docx]

**Supplemental Material. Simulate Result III**

The full results of CDRs for different conditions were presented in this supplemental material. The boxplots of AFIs for different conditions were presented in Figures S1 – S20. For convenient, let M1 = the SH-DINA model. M2 = the SH-DINO model, M3 = the SH-ACDM, and M4 = the SH-GDINA model.

Table S1. CDRs of RFIs for different models with *non-hierarchical* attribute structures.

| GM | CM | **Low item quality** | | | | | **High item quality** | | | | |
| --- | --- | --- | --- | --- | --- | --- | --- | --- | --- | --- | --- |
|  |  | AIC | BIC | aBIC | CAIC | AICc | AIC | BIC | aBIC | CAIC | AICc |
|  |  |  |  |  |  | *N* = 1,000 | |  |  |  |  |
| M1 | M1 | **1** | **1** | **1** | **1** | **1** | **1** | **1** | **1** | **1** | **1** |
|  | M2 | 0 | 0 | 0 | 0 | 0 | 0 | 0 | 0 | 0 | 0 |
|  | M3 | 0 | 0 | 0 | 0 | 0 | 0 | 0 | 0 | 0 | 0 |
|  | M4 | 0 | 0 | 0 | 0 | 0 | 0 | 0 | 0 | 0 | 0 |
| M2 | M1 | 0 | 0 | 0 | 0 | 0 | 0 | 0 | 0 | 0 | 0 |
|  | M2 | **1** | **1** | **1** | **1** | **1** | **1** | **1** | **1** | **1** | **1** |
|  | M3 | 0 | 0 | 0 | 0 | 0 | 0 | 0 | 0 | 0 | 0 |
|  | M4 | 0 | 0 | 0 | 0 | 0 | 0 | 0 | 0 | 0 | 0 |
| M3 | M1 | 0 | 0 | 0 | 0 | 0 | 0 | 0 | 0 | 0 | 0 |
|  | M2 | 0 | 0 | 0 | 0 | 0 | 0 | 0 | 0 | 0 | 0 |
|  | M3 | **1** | **1** | **1** | **1** | **1** | **1** | **1** | **1** | **1** | **1** |
|  | M4 | 0 | 0 | 0 | 0 | 0 | 0 | 0 | 0 | 0 | 0 |
| M4 | M1 | 0 | 0 | 0 | 0 | 0 | 0 | 0 | 0 | 0 | 0 |
|  | M2 | 0 | 0 | 0 | 0 | 0 | 0 | 0 | 0 | 0 | 0 |
|  | M3 | 0 | **1** | **.502** | **1** | .134 | 0 | 0 | 0 | 0 | 0 |
|  | M4 | **1** | 0 | .498 | 0 | **.866** | **1** | **1** | **1** | **1** | **1** |
|  |  |  |  |  |  | *N* = 3,000 | |  |  |  |  |
| M1 | M1 | **1** | **1** | **1** | **1** | **1** | **1** | **1** | **1** | **1** | **1** |
|  | M2 | 0 | 0 | 0 | 0 | 0 | 0 | 0 | 0 | 0 | 0 |
|  | M3 | 0 | 0 | 0 | 0 | 0 | 0 | 0 | 0 | 0 | 0 |
|  | M4 | 0 | 0 | 0 | 0 | 0 | 0 | 0 | 0 | 0 | 0 |
| M2 | M1 | 0 | 0 | 0 | 0 | 0 | 0 | 0 | 0 | 0 | 0 |
|  | M2 | **1** | **1** | **1** | **1** | **1** | **1** | **1** | **1** | **1** | **1** |
|  | M3 | 0 | 0 | 0 | 0 | 0 | 0 | 0 | 0 | 0 | 0 |
|  | M4 | 0 | 0 | 0 | 0 | 0 | 0 | 0 | 0 | 0 | 0 |
| M3 | M1 | 0 | 0 | 0 | 0 | 0 | 0 | 0 | 0 | 0 | 0 |
|  | M2 | 0 | 0 | 0 | 0 | 0 | 0 | 0 | 0 | 0 | 0 |
|  | M3 | **1** | **1** | **1** | **1** | **1** | **1** | **1** | **1** | **1** | **1** |
|  | M4 | 0 | 0 | 0 | 0 | 0 | 0 | 0 | 0 | 0 | 0 |
| M4 | M1 | 0 | 0 | 0 | 0 | 0 | 0 | 0 | 0 | 0 | 0 |
|  | M2 | 0 | 0 | 0 | 0 | 0 | 0 | 0 | 0 | 0 | 0 |
|  | M3 | 0 | **.76** | 0 | **.978** | 0 | 0 | 0 | 0 | 0 | 0 |
|  | M4 | **1** | .24 | **1** | .022 | **1** | **1** | **1** | **1** | **1** | **1** |

*Note*. GM = data generation model; CM = calibration model.

Table S2. CDRs of RFIs for different models with *linear* attribute structures.

| GM | CM | **Low item quality** | | | | | **High item quality** | | | | |
| --- | --- | --- | --- | --- | --- | --- | --- | --- | --- | --- | --- |
|  |  | AIC | BIC | aBIC | CAIC | AICc | AIC | BIC | aBIC | CAIC | AICc |
|  |  |  |  |  |  | *N* = 1,000 | |  |  |  |  |
| M1 | M1 | **1** | **1** | **1** | **1** | **1** | **1** | **1** | **1** | **1** | **1** |
|  | M2 | 0 | 0 | 0 | 0 | 0 | 0 | 0 | 0 | 0 | 0 |
|  | M3 | 0 | 0 | 0 | 0 | 0 | 0 | 0 | 0 | 0 | 0 |
|  | M4 | 0 | 0 | 0 | 0 | 0 | 0 | 0 | 0 | 0 | 0 |
| M2 | M1 | 0 | 0 | 0 | 0 | 0 | 0 | 0 | 0 | 0 | 0 |
|  | M2 | **1** | **1** | **1** | **1** | **1** | **.998** | **1** | **1** | **1** | **1** |
|  | M3 | 0 | 0 | 0 | 0 | 0 | 0 | 0 | 0 | 0 | 0 |
|  | M4 | 0 | 0 | 0 | 0 | 0 | .002 | 0 | 0 | 0 | 0 |
| M3 | M1 | 0 | 0 | 0 | 0 | 0 | 0 | 0 | 0 | 0 | 0 |
|  | M2 | 0 | 0 | 0 | 0 | 0 | 0 | 0 | 0 | 0 | 0 |
|  | M3 | .088 | .088 | .088 | .088 | .088 | .01 | .01 | .01 | .01 | .01 |
|  | M4 | **.912** | **.912** | **.912** | **.912** | **.912** | **.99** | **.99** | **.99** | **.99** | **.99** |
| M4 | M1 | 0 | 0 | 0 | 0 | 0 | 0 | 0 | 0 | 0 | 0 |
|  | M2 | 0 | 0 | 0 | 0 | 0 | 0 | 0 | 0 | 0 | 0 |
|  | M3 | .126 | .126 | .126 | .126 | .126 | .01 | .01 | .01 | .01 | .01 |
|  | M4 | **.874** | **.874** | **.874** | **.874** | **.874** | **.99** | **.99** | **.99** | **.99** | **.99** |
|  |  |  |  |  |  | *N* = 3,000 | |  |  |  |  |
| M1 | M1 | **1** | **1** | **1** | **1** | **1** | **1** | **1** | **1** | **1** | **1** |
|  | M2 | 0 | 0 | 0 | 0 | 0 | 0 | 0 | 0 | 0 | 0 |
|  | M3 | 0 | 0 | 0 | 0 | 0 | 0 | 0 | 0 | 0 | 0 |
|  | M4 | 0 | 0 | 0 | 0 | 0 | 0 | 0 | 0 | 0 | 0 |
| M2 | M1 | 0 | 0 | 0 | 0 | 0 | 0 | 0 | 0 | 0 | 0 |
|  | M2 | **1** | **1** | **1** | **1** | **1** | **1** | **1** | **1** | **1** | **1** |
|  | M3 | 0 | 0 | 0 | 0 | 0 | 0 | 0 | 0 | 0 | 0 |
|  | M4 | 0 | 0 | 0 | 0 | 0 | 0 | 0 | 0 | 0 | 0 |
| M3 | M1 | 0 | 0 | 0 | 0 | 0 | 0 | 0 | 0 | 0 | 0 |
|  | M2 | 0 | 0 | 0 | 0 | 0 | 0 | 0 | 0 | 0 | 0 |
|  | M3 | .01 | .01 | .01 | .01 | .01 | .016 | .016 | .016 | .016 | .016 |
|  | M4 | **.99** | **.99** | **.99** | **.99** | **.99** | **.984** | **.984** | **.984** | **.984** | **.984** |
| M4 | M1 | 0 | 0 | 0 | 0 | 0 | 0 | 0 | 0 | 0 | 0 |
|  | M2 | 0 | 0 | 0 | 0 | 0 | 0 | 0 | 0 | 0 | 0 |
|  | M3 | .168 | .168 | .168 | .168 | .168 | .006 | .006 | .006 | .006 | .006 |
|  | M4 | **.832** | **.832** | **.832** | **.832** | **.832** | **.994** | **.994** | **.994** | **.994** | **.994** |

*Note*. GM = data generation model; CM = calibration model.

Table S3. CDRs of RFIs for different models with *convergent* attribute structures.

| GM | CM | **Low item quality** | | | | | **High item quality** | | | | |
| --- | --- | --- | --- | --- | --- | --- | --- | --- | --- | --- | --- |
|  |  | AIC | BIC | aBIC | CAIC | AICc | AIC | BIC | aBIC | CAIC | AICc |
|  |  |  |  |  |  | *N* = 1,000 | |  |  |  |  |
| M1 | M1 | **1** | **1** | **1** | **1** | **1** | **1** | **1** | **1** | **1** | **1** |
|  | M2 | 0 | 0 | 0 | 0 | 0 | 0 | 0 | 0 | 0 | 0 |
|  | M3 | 0 | 0 | 0 | 0 | 0 | 0 | 0 | 0 | 0 | 0 |
|  | M4 | 0 | 0 | 0 | 0 | 0 | 0 | 0 | 0 | 0 | 0 |
| M2 | M1 | 0 | 0 | 0 | 0 | 0 | 0 | 0 | 0 | 0 | 0 |
|  | M2 | **1** | **1** | **1** | **1** | **1** | **.998** | **1** | **1** | **1** | **1** |
|  | M3 | 0 | 0 | 0 | 0 | 0 | 0 | 0 | 0 | 0 | 0 |
|  | M4 | 0 | 0 | 0 | 0 | 0 | .002 | 0 | 0 | 0 | 0 |
| M3 | M1 | 0 | 0 | 0 | 0 | 0 | 0 | 0 | 0 | 0 | 0 |
|  | M2 | 0 | 0 | 0 | 0 | 0 | 0 | 0 | 0 | 0 | 0 |
|  | M3 | **.97** | **1** | **1** | **1** | **1** | **.938** | **1** | **.998** | **1** | **.998** |
|  | M4 | .03 | 0 | 0 | 0 | 0 | .062 | 0 | .002 | 0 | .002 |
| M4 | M1 | 0 | 0 | 0 | 0 | 0 | 0 | 0 | 0 | 0 | 0 |
|  | M2 | 0 | 0 | 0 | 0 | 0 | 0 | 0 | 0 | 0 | 0 |
|  | M3 | 0 | **.642** | .05 | **.822** | .014 | 0 | .046 | 0 | .126 | 0 |
|  | M4 | **1** | .358 | **.95** | .178 | **.986** | **1** | **.954** | **1** | **.874** | **1** |
|  |  |  |  |  |  | *N* = 3,000 | |  |  |  |  |
| M1 | M1 | **1** | **1** | **1** | **1** | **1** | **1** | **1** | **1** | **1** | **1** |
|  | M2 | 0 | 0 | 0 | 0 | 0 | 0 | 0 | 0 | 0 | 0 |
|  | M3 | 0 | 0 | 0 | 0 | 0 | 0 | 0 | 0 | 0 | 0 |
|  | M4 | 0 | 0 | 0 | 0 | 0 | 0 | 0 | 0 | 0 | 0 |
| M2 | M1 | 0 | 0 | 0 | 0 | 0 | 0 | 0 | 0 | 0 | 0 |
|  | M2 | **1** | **1** | **1** | **1** | **1** | **1** | **1** | **1** | **1** | **1** |
|  | M3 | 0 | 0 | 0 | 0 | 0 | 0 | 0 | 0 | 0 | 0 |
|  | M4 | 0 | 0 | 0 | 0 | 0 | 0 | 0 | 0 | 0 | 0 |
| M3 | M1 | 0 | 0 | 0 | 0 | 0 | 0 | 0 | 0 | 0 | 0 |
|  | M2 | 0 | 0 | 0 | 0 | 0 | 0 | 0 | 0 | 0 | 0 |
|  | M3 | **.966** | **1** | **1** | **1** | **.988** | **.954** | **1** | **1** | **1** | **.97** |
|  | M4 | .034 | 0 | 0 | 0 | .012 | .046 | 0 | 0 | 0 | .03 |
| M4 | M1 | 0 | 0 | 0 | 0 | 0 | 0 | 0 | 0 | 0 | 0 |
|  | M2 | 0 | 0 | 0 | 0 | 0 | 0 | 0 | 0 | 0 | 0 |
|  | M3 | 0 | .006 | 0 | .032 | 0 | 0 | 0 | 0 | 0 | 0 |
|  | M4 | **1** | **.994** | **1** | **.968** | **1** | **1** | **1** | **1** | **1** | **1** |

*Note*. GM = data generation model; CM = calibration model.

Table S4. CDRs of RFIs for different models with *divergent* attribute structures.

| GM | CM | **Low item quality** | | | | | **High item quality** | | | | |
| --- | --- | --- | --- | --- | --- | --- | --- | --- | --- | --- | --- |
|  |  | AIC | BIC | aBIC | CAIC | AICc | AIC | BIC | aBIC | CAIC | AICc |
|  |  |  |  |  |  | *N* = 1,000 | |  |  |  |  |
| M1 | M1 | **1** | **1** | **1** | **1** | **1** | **1** | **1** | **1** | **1** | **1** |
|  | M2 | 0 | 0 | 0 | 0 | 0 | 0 | 0 | 0 | 0 | 0 |
|  | M3 | 0 | 0 | 0 | 0 | 0 | 0 | 0 | 0 | 0 | 0 |
|  | M4 | 0 | 0 | 0 | 0 | 0 | 0 | 0 | 0 | 0 | 0 |
| M2 | M1 | 0 | 0 | 0 | 0 | 0 | 0 | 0 | 0 | 0 | 0 |
|  | M2 | **1** | **1** | **1** | **1** | **1** | **1** | **1** | **1** | **1** | **1** |
|  | M3 | 0 | 0 | 0 | 0 | 0 | 0 | 0 | 0 | 0 | 0 |
|  | M4 | 0 | 0 | 0 | 0 | 0 | 0 | 0 | 0 | 0 | 0 |
| M3 | M1 | 0 | 0 | 0 | 0 | 0 | 0 | 0 | 0 | 0 | 0 |
|  | M2 | 0 | 0 | 0 | 0 | 0 | 0 | 0 | 0 | 0 | 0 |
|  | M3 | **.996** | **1** | **1** | **1** | **1** | **.994** | **1** | **1** | **1** | **1** |
|  | M4 | .004 | 0 | 0 | 0 | 0 | .006 | 0 | 0 | 0 | 0 |
| M4 | M1 | 0 | 0 | 0 | 0 | 0 | 0 | 0 | 0 | 0 | 0 |
|  | M2 | 0 | 0 | 0 | 0 | 0 | 0 | 0 | 0 | 0 | 0 |
|  | M3 | 0 | **.888** | .02 | **.988** | 0 | 0 | .062 | 0 | .236 | 0 |
|  | M4 | **1** | .112 | **.98** | .012 | **1** | **1** | **.938** | **1** | **.764** | **1** |
|  |  |  |  |  |  | *N* = 3,000 | |  |  |  |  |
| M1 | M1 | **1** | **1** | **1** | **1** | **1** | **1** | **1** | **1** | **1** | **1** |
|  | M2 | 0 | 0 | 0 | 0 | 0 | 0 | 0 | 0 | 0 | 0 |
|  | M3 | 0 | 0 | 0 | 0 | 0 | 0 | 0 | 0 | 0 | 0 |
|  | M4 | 0 | 0 | 0 | 0 | 0 | 0 | 0 | 0 | 0 | 0 |
| M2 | M1 | 0 | 0 | 0 | 0 | 0 | 0 | 0 | 0 | 0 | 0 |
|  | M2 | **1** | **1** | **1** | **1** | **1** | **1** | **1** | **1** | **1** | **1** |
|  | M3 | 0 | 0 | 0 | 0 | 0 | 0 | 0 | 0 | 0 | 0 |
|  | M4 | 0 | 0 | 0 | 0 | 0 | 0 | 0 | 0 | 0 | 0 |
| M3 | M1 | 0 | 0 | 0 | 0 | 0 | 0 | 0 | 0 | 0 | 0 |
|  | M2 | 0 | 0 | 0 | 0 | 0 | 0 | 0 | 0 | 0 | 0 |
|  | M3 | **.992** | **1** | **1** | **1** | **1** | **.992** | **1** | **1** | **1** | **.998** |
|  | M4 | .008 | 0 | 0 | 0 | 0 | .008 | 0 | 0 | 0 | .002 |
| M4 | M1 | 0 | 0 | 0 | 0 | 0 | 0 | 0 | 0 | 0 | 0 |
|  | M2 | 0 | 0 | 0 | 0 | 0 | 0 | 0 | 0 | 0 | 0 |
|  | M3 | 0 | 0 | 0 | .056 | 0 | 0 | 0 | 0 | 0 | 0 |
|  | M4 | **1** | **1** | **1** | **.944** | **1** | **1** | **1** | **1** | **1** | **1** |

*Note*. GM = data generation model; CM = calibration model.

Table S5. CDRs of RFIs for different models with *unstructured* attribute structures.

| GM | CM | **Low item quality** | | | | | **High item quality** | | | | |
| --- | --- | --- | --- | --- | --- | --- | --- | --- | --- | --- | --- |
|  |  | AIC | BIC | aBIC | CAIC | AICc | AIC | BIC | aBIC | CAIC | AICc |
|  |  |  |  |  |  | *N* = 1,000 | |  |  |  |  |
| M1 | M1 | **1** | **1** | **1** | **1** | **1** | **1** | **1** | **1** | **1** | **1** |
|  | M2 | 0 | 0 | 0 | 0 | 0 | 0 | 0 | 0 | 0 | 0 |
|  | M3 | 0 | 0 | 0 | 0 | 0 | 0 | 0 | 0 | 0 | 0 |
|  | M4 | 0 | 0 | 0 | 0 | 0 | 0 | 0 | 0 | 0 | 0 |
| M2 | M1 | 0 | 0 | 0 | 0 | 0 | 0 | 0 | 0 | 0 | 0 |
|  | M2 | **1** | **1** | **1** | **1** | **1** | **1** | **1** | **1** | **1** | **1** |
|  | M3 | 0 | 0 | 0 | 0 | 0 | 0 | 0 | 0 | 0 | 0 |
|  | M4 | 0 | 0 | 0 | 0 | 0 | 0 | 0 | 0 | 0 | 0 |
| M3 | M1 | 0 | 0 | 0 | 0 | 0 | 0 | 0 | 0 | 0 | 0 |
|  | M2 | 0 | 0 | 0 | 0 | 0 | 0 | 0 | 0 | 0 | 0 |
|  | M3 | **1** | **1** | **1** | **1** | **1** | **.996** | **1** | **1** | **1** | **1** |
|  | M4 | 0 | 0 | 0 | 0 | 0 | .004 | 0 | 0 | 0 | 0 |
| M4 | M1 | 0 | 0 | 0 | 0 | 0 | 0 | 0 | 0 | 0 | 0 |
|  | M2 | 0 | 0 | 0 | 0 | 0 | 0 | 0 | 0 | 0 | 0 |
|  | M3 | 0 | **.894** | .002 | **.998** | 0 | 0 | 0 | 0 | .018 | 0 |
|  | M4 | **1** | .106 | **.998** | .002 | **1** | **1** | **1** | **1** | **.982** | **1** |
|  |  |  |  |  |  | *N* = 3,000 | |  |  |  |  |
| M1 | M1 | **1** | **1** | **1** | **1** | **1** | **1** | **1** | **1** | **1** | **1** |
|  | M2 | 0 | 0 | 0 | 0 | 0 | 0 | 0 | 0 | 0 | 0 |
|  | M3 | 0 | 0 | 0 | 0 | 0 | 0 | 0 | 0 | 0 | 0 |
|  | M4 | 0 | 0 | 0 | 0 | 0 | 0 | 0 | 0 | 0 | 0 |
| M2 | M1 | 0 | 0 | 0 | 0 | 0 | 0 | 0 | 0 | 0 | 0 |
|  | M2 | **1** | **1** | **1** | **1** | **1** | **1** | **1** | **1** | **1** | **1** |
|  | M3 | 0 | 0 | 0 | 0 | 0 | 0 | 0 | 0 | 0 | 0 |
|  | M4 | 0 | 0 | 0 | 0 | 0 | 0 | 0 | 0 | 0 | 0 |
| M3 | M1 | 0 | 0 | 0 | 0 | 0 | 0 | 0 | 0 | 0 | 0 |
|  | M2 | 0 | 0 | 0 | 0 | 0 | 0 | 0 | 0 | 0 | 0 |
|  | M3 | **1** | **1** | **1** | **1** | **1** | **1** | **1** | **1** | **1** | **1** |
|  | M4 | **0** | **0** | **0** | **0** | **0** | **0** | **0** | **0** | **0** | **0** |
| M4 | M1 | 0 | 0 | 0 | 0 | 0 | 0 | 0 | 0 | 0 | 0 |
|  | M2 | 0 | 0 | 0 | 0 | 0 | 0 | 0 | 0 | 0 | 0 |
|  | M3 | 0 | **.652** | .002 | **.938** | 0 | 0 | 0 | 0 | 0 | 0 |
|  | M4 | **1** | .348 | **.998** | .062 | **1** | **1** | **1** | **1** | **1** | **1** |

*Note*. GM = data generation model; CM = calibration model.

Table S6. CDRs of AFIs for different models with *non-hierarchical* attribute structures.

| GM | CM | **Low item quality** | | | **High item quality** | | |
| --- | --- | --- | --- | --- | --- | --- | --- |
|  |  | MADcor | 100MAD  RESIDCOV | SRMSR | MADcor | 100MAD  RESIDCOV | SRMSR |
|  |  |  |  | *N* = 1,000 | |  |  |
| M1 | M1 | .064 | .088 | .048 | .056 | .078 | .046 |
|  | M2 | 0 | 0 | 0 | 0 | 0 | 0 |
|  | M3 | 0 | 0 | 0 | 0 | 0 | 0 |
|  | M4 | **.936** | **.912** | **.952** | **.944** | **.922** | **.954** |
| M2 | M1 | 0 | 0 | 0 | 0 | 0 | 0 |
|  | M2 | .044 | .052 | .03 | .036 | .044 | .028 |
|  | M3 | .032 | .01 | .01 | 0 | 0 | 0 |
|  | M4 | **.924** | **.938** | **.96** | **.964** | **.956** | **.972** |
| M3 | M1 | 0 | 0 | 0 | 0 | 0 | 0 |
|  | M2 | 0 | 0 | 0 | 0 | 0 | 0 |
|  | M3 | .482 | .458 | .476 | .292 | .254 | .274 |
|  | M4 | **.518** | **.542** | **.524** | **.708** | **.746** | **.726** |
| M4 | M1 | 0 | 0 | 0 | 0 | 0 | 0 |
|  | M2 | 0 | 0 | 0 | 0 | 0 | 0 |
|  | M3 | .21 | .186 | .2 | .008 | 0 | .006 |
|  | M4 | **.79** | **.814** | **.8** | **.992** | **1** | **.994** |
|  |  |  |  | *N* = 3,000 | |  |  |
| M1 | M1 | .044 | .076 | .038 | .058 | .064 | .036 |
|  | M2 | 0 | 0 | 0 | 0 | 0 | 0 |
|  | M3 | 0 | 0 | 0 | 0 | 0 | 0 |
|  | M4 | **0.956** | **.924** | **.962** | **.942** | **.936** | **.964** |
| M2 | M1 | 0 | 0 | 0 | 0 | 0 | 0 |
|  | M2 | .03 | .044 | .012 | .03 | .042 | .024 |
|  | M3 | 0 | 0 | 0 | 0 | 0 | 0 |
|  | M4 | **.97** | **.956** | **.988** | **.97** | **.958** | **.976** |
| M3 | M1 | 0 | 0 | 0 | 0 | 0 | 0 |
|  | M2 | 0 | 0 | 0 | 0 | 0 | 0 |
|  | M3 | .426 | .394 | .386 | .244 | .2 | .24 |
|  | M4 | **.574** | **.606** | **.614** | **.756** | **.8** | **.76** |
| M4 | M1 | 0 | 0 | 0 | 0 | 0 | 0 |
|  | M2 | 0 | 0 | 0 | 0 | 0 | 0 |
|  | M3 | .156 | .102 | .12 | 0 | 0 | 0 |
|  | M4 | **.844** | **.898** | **.88** | **1** | **1** | **1** |

*Note*. GM = data generation model; CM = calibration model.

Table S7. CDRs of AFIs for different models with *linear* attribute structures.

| GM | CM | **Low item quality** | | | **High item quality** | | |
| --- | --- | --- | --- | --- | --- | --- | --- |
|  |  | MADcor | 100MAD  RESIDCOV | SRMSR | MADcor | 100MAD  RESIDCOV | SRMSR |
|  |  |  |  | *N* = 1,000 | |  |  |
| M1 | M1 | .158 | .142 | .114 | .176 | .144 | .126 |
|  | M2 | 0 | 0 | 0 | 0 | 0 | 0 |
|  | M3 | **.53** | **.526** | **.528** | **.42** | **.472** | **.454** |
|  | M4 | .312 | .332 | .358 | .404 | .384 | .42 |
| M2 | M1 | 0 | 0 | 0 | 0 | 0 | 0 |
|  | M2 | .082 | .082 | .056 | .042 | .044 | .036 |
|  | M3 | .444 | .438 | .454 | **.536** | **.522** | **.514** |
|  | M4 | **.474** | **.48** | **.49** | .422 | .434 | .45 |
| M3 | M1 | 0 | 0 | 0 | 0 | 0 | 0 |
|  | M2 | 0 | 0 | 0 | 0 | 0 | 0 |
|  | M3 | **.554** | **.548** | **.558** | **.554** | **.592** | **.54** |
|  | M4 | .446 | .452 | .442 | .446 | .408 | .46 |
| M4 | M1 | 0 | 0 | 0 | 0 | 0 | 0 |
|  | M2 | 0 | 0 | 0 | 0 | 0 | 0 |
|  | M3 | .46 | .492 | .492 | **.562** | **.568** | **.556** |
|  | M4 | **.54** | **.508** | **.508** | .438 | .432 | .444 |
|  |  |  |  | *N* = 3,000 | |  |  |
| M1 | M1 | .146 | .158 | .114 | .154 | .162 | .128 |
|  | M2 | 0 | 0 | 0 | 0 | 0 | 0 |
|  | M3 | **.438** | **.44** | **.488** | **.464** | **.44** | **.508** |
|  | M4 | .416 | .402 | .398 | .382 | .398 | .364 |
| M2 | M1 | 0 | 0 | 0 | 0 | 0 | 0 |
|  | M2 | .078 | .072 | .048 | .078 | .072 | .048 |
|  | M3 | .422 | .438 | .46 | .422 | .438 | .46 |
|  | M4 | **.5** | **.49** | **.492** | **.5** | **.49** | **.492** |
| M3 | M1 | 0 | 0 | 0 | 0 | 0 | 0 |
|  | M2 | 0 | 0 | 0 | 0 | 0 | 0 |
|  | M3 | .492 | .462 | .48 | **.502** | .498 | **.53** |
|  | M4 | **.508** | **.538** | **.52** | .498 | **.502** | .47 |
| M4 | M1 | 0 | 0 | 0 | 0 | 0 | 0 |
|  | M2 | 0 | 0 | 0 | 0 | 0 | 0 |
|  | M3 | **.558** | .49 | **.554** | **.562** | **.542** | **.544** |
|  | M4 | .442 | .51 | .446 | .438 | .458 | .456 |

*Note*. GM = data generation model; CM = calibration model.

Table S8. CDRs of AFIs for different models with *convergent* attribute structures.

| GM | CM | **Low item quality** | | | **High item quality** | | |
| --- | --- | --- | --- | --- | --- | --- | --- |
|  |  | MADcor | 100MAD  RESIDCOV | SRMSR | MADcor | 100MAD  RESIDCOV | SRMSR |
|  |  |  |  | *N* = 1,000 | |  |  |
| M1 | M1 | .120 | .130 | .104 | .112 | .126 | .1 |
|  | M2 | 0 | 0 | 0 | 0 | 0 | 0 |
|  | M3 | .006 | .002 | .004 | 0 | 0 | 0 |
|  | M4 | **.874** | **.868** | **.892** | **.888** | **.874** | **.9** |
| M2 | M1 | 0 | 0 | 0 | 0 | 0 | 0 |
|  | M2 | .088 | .088 | .056 | .074 | .074 | .056 |
|  | M3 | .058 | .038 | .050 | 0 | 0 | 0 |
|  | M4 | **.854** | **.874** | **.894** | **.926** | **.926** | **.944** |
| M3 | M1 | 0 | 0 | 0 | 0 | 0 | 0 |
|  | M2 | 0 | 0 | 0 | 0 | 0 | 0 |
|  | M3 | .436 | .44 | .434 | .39 | .342 | .368 |
|  | M4 | **.564** | **.56** | **.566** | **.61** | **.658** | **.632** |
| M4 | M1 | 0 | 0 | 0 | 0 | 0 | 0 |
|  | M2 | 0 | 0 | 0 | 0 | 0 | 0 |
|  | M3 | .144 | .12 | .11 | .13 | .098 | .114 |
|  | M4 | **.856** | **.88** | **.89** | **.87** | **.902** | **.886** |
|  |  |  |  | *N* = 3,000 | |  |  |
| M1 | M1 | .168 | .178 | .12 | .146 | .164 | .116 |
|  | M2 | 0 | 0 | 0 | 0 | 0 | 0 |
|  | M3 | 0 | 0 | 0 | 0 | 0 | 0 |
|  | M4 | **.832** | **.822** | **.88** | **.854** | **.836** | **.884** |
| M2 | M1 | 0 | 0 | 0 | 0 | 0 | 0 |
|  | M2 | .076 | .076 | .04 | .04 | .044 | .024 |
|  | M3 | 0 | 0 | 0 | 0 | 0 | 0 |
|  | M4 | **.924** | **.924** | **.96** | **.96** | **.956** | **.976** |
| M3 | M1 | 0 | 0 | 0 | 0 | 0 | 0 |
|  | M2 | 0 | 0 | 0 | 0 | 0 | 0 |
|  | M3 | .436 | .41 | .426 | .382 | .352 | .378 |
|  | M4 | **.564** | **.59** | **.574** | **.618** | **.648** | **.622** |
| M4 | M1 | 0 | 0 | 0 | 0 | 0 | 0 |
|  | M2 | 0 | 0 | 0 | 0 | 0 | 0 |
|  | M3 | .144 | .104 | .122 | .112 | .088 | .094 |
|  | M4 | **.856** | **.896** | **.878** | **.888** | **.912** | **.906** |

*Note*. GM = data generation model; CM = calibration model.

Table S9. CDRs of AFIs for different models with *divergent* attribute structures.

| GM | CM | **Low item quality** | | | **High item quality** | | |
| --- | --- | --- | --- | --- | --- | --- | --- |
|  |  | MADcor | 100MAD  RESIDCOV | SRMSR | MADcor | 100MAD  RESIDCOV | SRMSR |
|  |  |  |  | *N* = 1,000 | |  |  |
| M1 | M1 | .146 | .138 | .094 | .084 | .082 | .066 |
|  | M2 | 0 | 0 | 0 | 0 | 0 | 0 |
|  | M3 | 0 | 0 | 0 | 0 | 0 | 0 |
|  | M4 | **.854** | **.862** | **.906** | **.916** | **.918** | **.934** |
| M2 | M1 | 0 | 0 | 0 | 0 | 0 | 0 |
|  | M2 | .082 | .086 | .058 | .04 | .038 | .024 |
|  | M3 | 0 | 0 | 0 | 0 | 0 | 0 |
|  | M4 | **.918** | **.914** | **.942** | **.96** | **.962** | **.976** |
| M3 | M1 | 0 | 0 | 0 | 0 | 0 | 0 |
|  | M2 | 0 | 0 | 0 | 0 | 0 | 0 |
|  | M3 | .446 | .42 | .414 | .35 | .306 | .314 |
|  | M4 | **.554** | **.58** | **.586** | **.65** | **.694** | **.686** |
| M4 | M1 | 0 | 0 | 0 | 0 | 0 | 0 |
|  | M2 | 0 | 0 | 0 | 0 | 0 | 0 |
|  | M3 | .198 | .082 | .178 | .146 | .102 | .112 |
|  | M4 | **.802** | **.918** | **.822** | **.854** | **.898** | **.888** |
|  |  |  |  | *N* = 3,000 | |  |  |
| M1 | M1 | .092 | .084 | .058 | .08 | .092 | .064 |
|  | M2 | 0 | 0 | 0 | 0 | 0 | 0 |
|  | M3 | 0 | 0 | 0 | 0 | 0 | 0 |
|  | M4 | **.908** | **.916** | **.942** | **.92** | **.908** | **.936** |
| M2 | M1 | 0 | 0 | 0 | 0 | 0 | 0 |
|  | M2 | .056 | .06 | .028 | .034 | .038 | .02 |
|  | M3 | 0 | 0 | 0 | 0 | 0 | 0 |
|  | M4 | **.944** | **.94** | **.972** | **.966** | **.962** | **.98** |
| M3 | M1 | 0 | 0 | 0 | 0 | 0 | 0 |
|  | M2 | 0 | 0 | 0 | 0 | 0 | 0 |
|  | M3 | .334 | .318 | .338 | .342 | .292 | .304 |
|  | M4 | **.666** | **.682** | **.662** | **.658** | **.708** | **.696** |
| M4 | M1 | 0 | 0 | 0 | 0 | 0 | 0 |
|  | M2 | 0 | 0 | 0 | 0 | 0 | 0 |
|  | M3 | .04 | .03 | .014 | 0 | 0 | 0 |
|  | M4 | **.96** | **.97** | **.986** | **1** | **1** | **1** |

*Note*. GM = data generation model; CM = calibration model.

Table S10. CDRs of AFIs for different models with *unstructured* attribute structures.

| GM | CM | **Low item quality** | | | **High item quality** | | |
| --- | --- | --- | --- | --- | --- | --- | --- |
|  |  | MADcor | 100MAD  RESIDCOV | SRMSR | MADcor | 100MAD  RESIDCOV | SRMSR |
|  |  |  |  | *N* = 1,000 | |  |  |
| M1 | M1 | .122 | .136 | .096 | .056 | .078 | .048 |
|  | M2 | 0 | 0 | 0 | 0 | 0 | 0 |
|  | M3 | 0 | 0 | 0 | 0 | 0 | 0 |
|  | M4 | **.878** | **.864** | **.904** | **.944** | **.922** | **.952** |
| M2 | M1 | 0 | 0 | 0 | 0 | 0 | 0 |
|  | M2 | .068 | .078 | .052 | .046 | .046 | .03 |
|  | M3 | .01 | .004 | .002 | 0 | 0 | 0 |
|  | M4 | **.922** | **.918** | **.946** | **.954** | **.954** | **.97** |
| M3 | M1 | 0 | 0 | 0 | 0 | 0 | 0 |
|  | M2 | 0 | 0 | 0 | 0 | 0 | 0 |
|  | M3 | .444 | .37 | .454 | .32 | .274 | .278 |
|  | M4 | **.556** | **.63** | **.546** | **.68** | **.726** | **.722** |
| M4 | M1 | 0 | 0 | 0 | 0 | 0 | 0 |
|  | M2 | 0 | 0 | 0 | 0 | 0 | 0 |
|  | M3 | .154 | .08 | .096 | .012 | .006 | .004 |
|  | M4 | **.846** | **.92** | **.904** | **.988** | **.994** | **.996** |
|  |  |  |  | *N* = 3,000 | |  |  |
| M1 | M1 | .058 | .08 | .054 | .064 | .086 | .054 |
|  | M2 | 0 | 0 | 0 | 0 | 0 | 0 |
|  | M3 | 0 | 0 | 0 | 0 | 0 | 0 |
|  | M4 | **.942** | **.92** | **.946** | **.936** | **.914** | **.946** |
| M2 | M1 | .058 | .08 | .054 | 0 | 0 | 0 |
|  | M2 | 0 | 0 | 0 | .05 | .052 | .046 |
|  | M3 | 0 | 0 | 0 | 0 | 0 | 0 |
|  | M4 | **.942** | **.92** | **.946** | **.95** | **.948** | **.954** |
| M3 | M1 | 0 | 0 | 0 | 0 | 0 | 0 |
|  | M2 | 0 | 0 | 0 | 0 | 0 | 0 |
|  | M3 | .398 | .352 | .376 | .29 | .274 | .264 |
|  | M4 | **.602** | **.648** | **.624** | **.71** | **.726** | **.736** |
| M4 | M1 | 0 | 0 | 0 | 0 | 0 | 0 |
|  | M2 | 0 | 0 | 0 | 0 | 0 | 0 |
|  | M3 | .174 | .084 | .112 | 0 | 0 | 0 |
|  | M4 | **.826** | **.916** | **.888** | **1** | **1** | **1** |

*Note*. GM = data generation model; CM = calibration model.


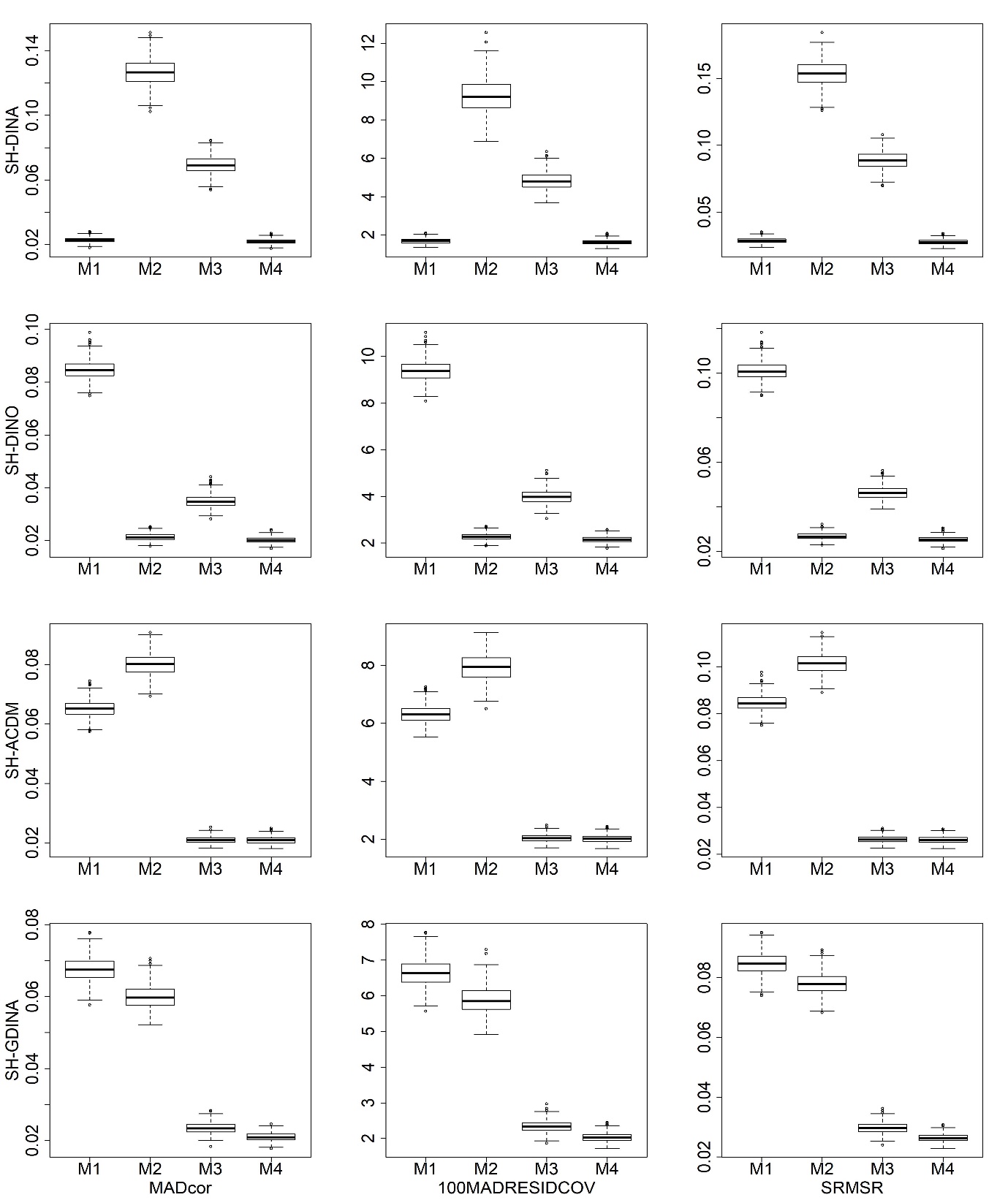


Figure S1. Boxplots of AFIs for *non-hierarchical* attribute structures

with small sample size in high item quality cases


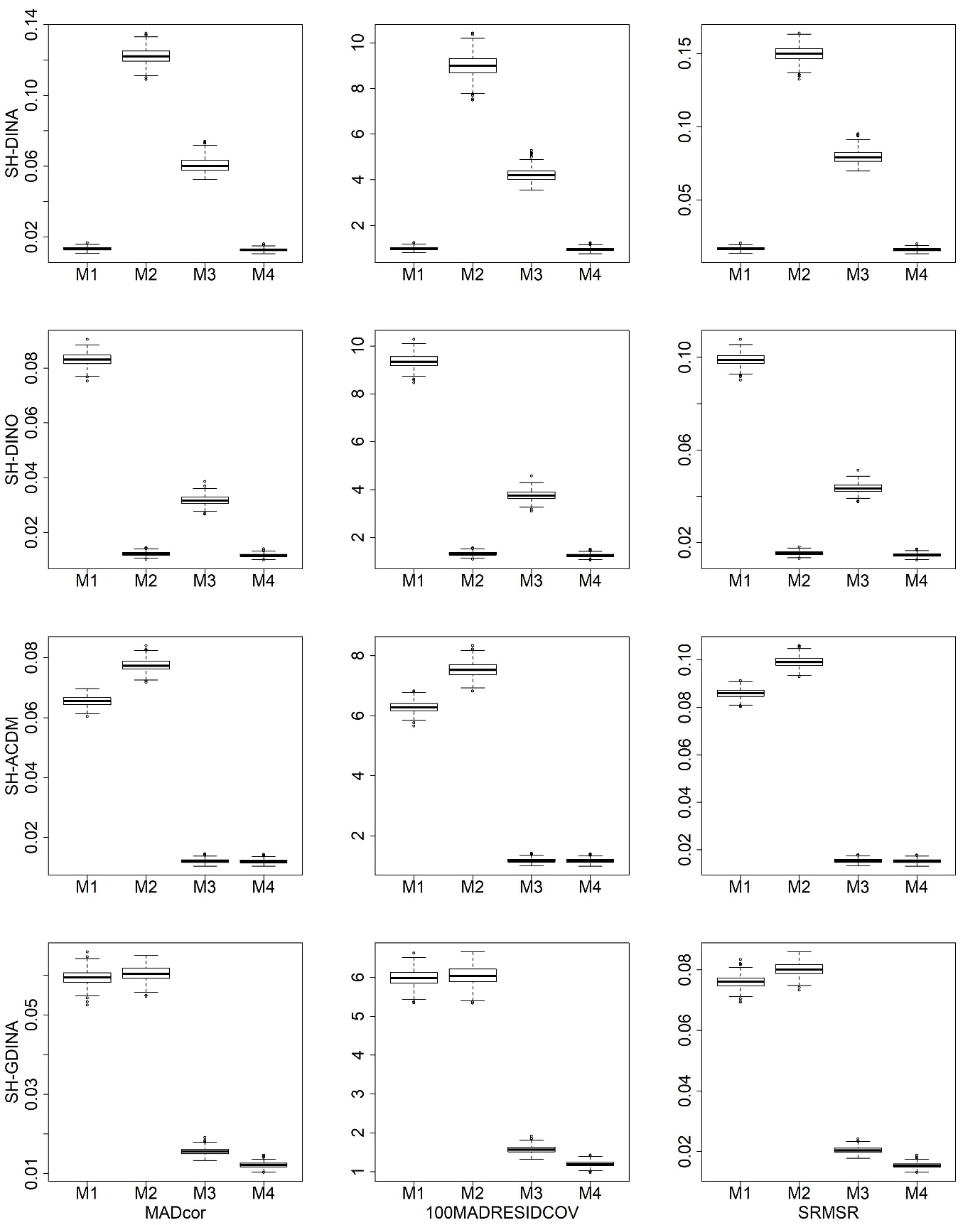


Figure S2. Boxplots of AFIs for *non-hierarchical* attribute structures

with large sample size in high item quality cases


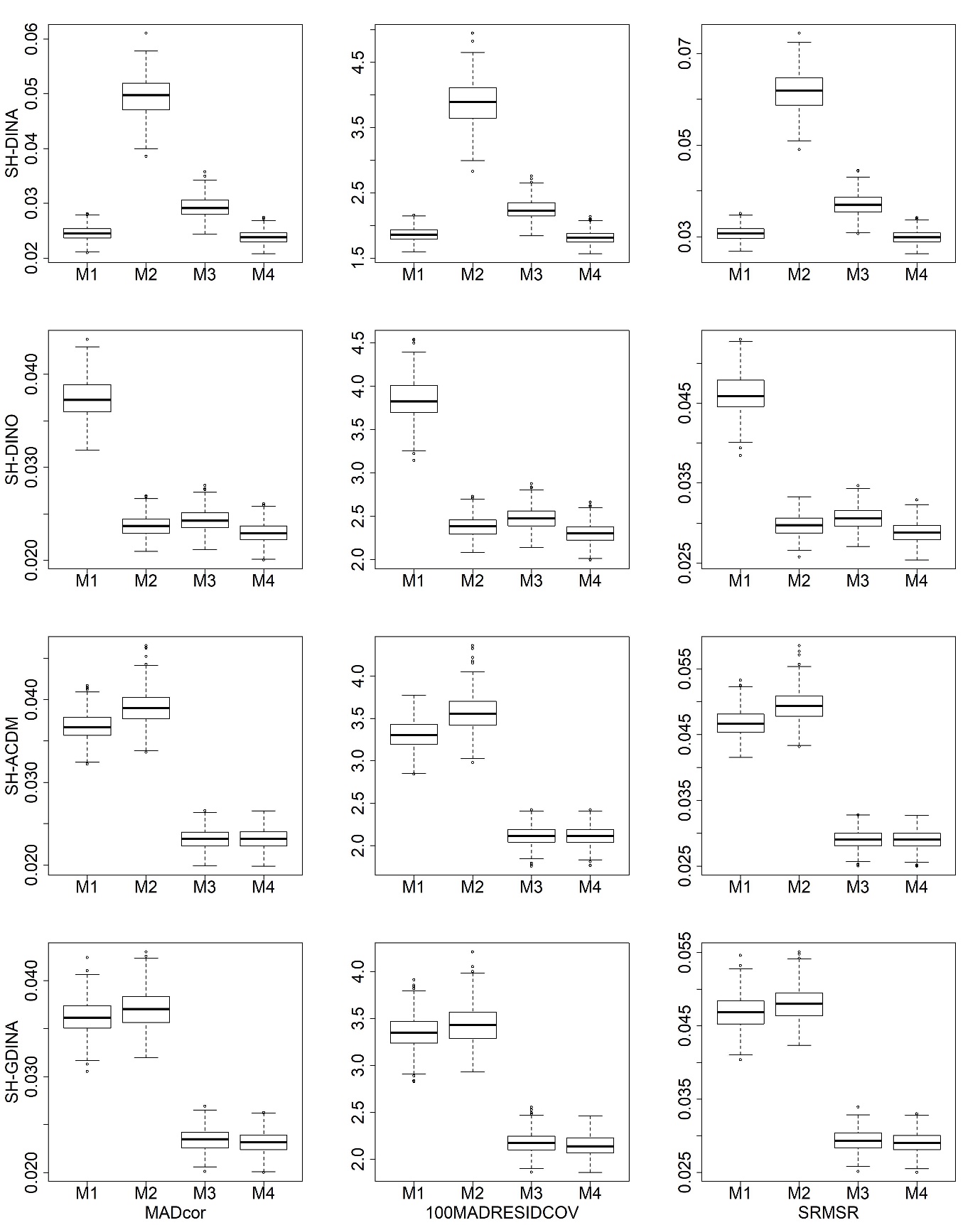


Figure S3. Boxplots of AFIs for *non-hierarchical* attribute structures

with small sample size in low item quality cases


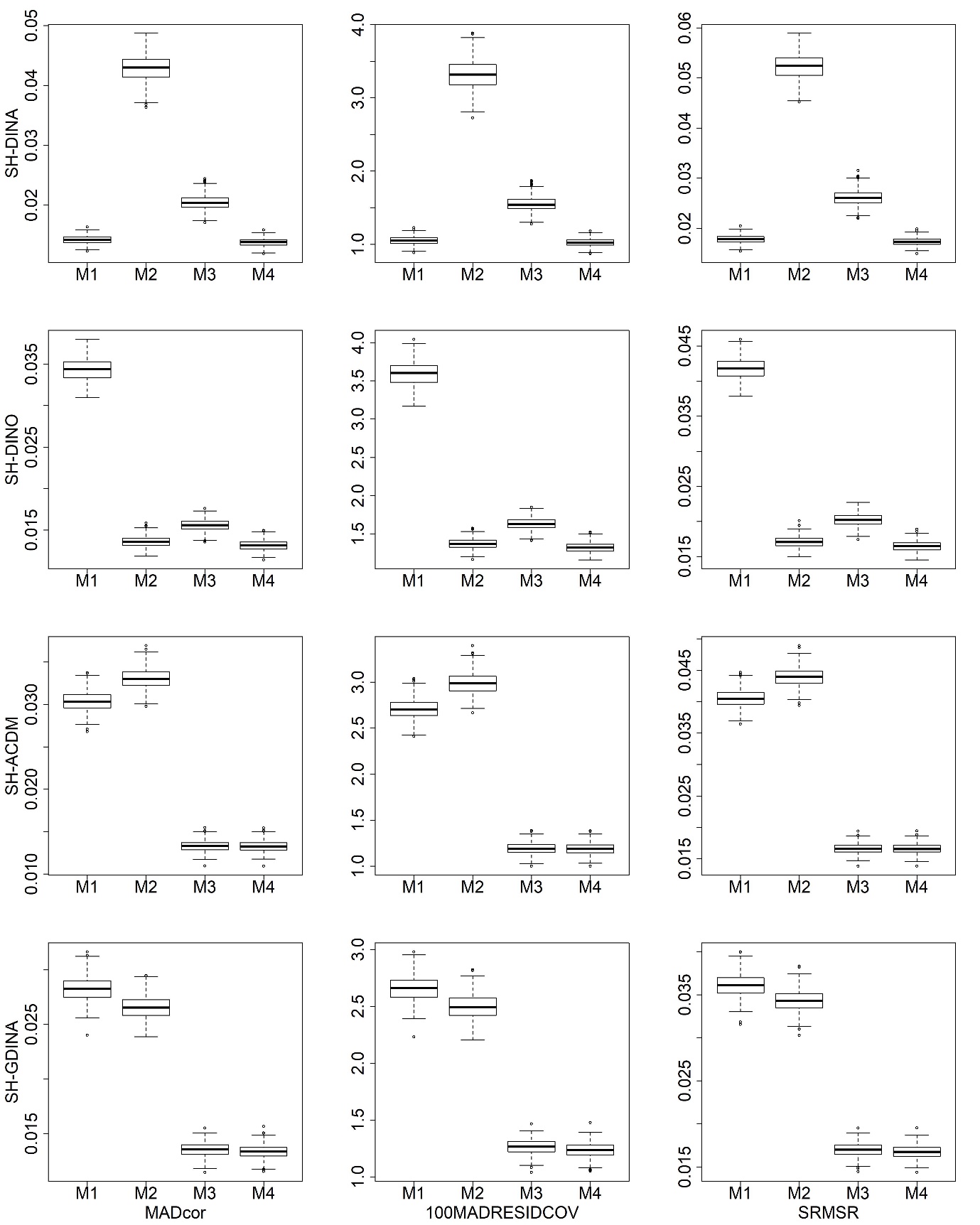


Figure S4. Boxplots of AFIs for *non-hierarchical* attribute structures

with large sample size in low item quality cases


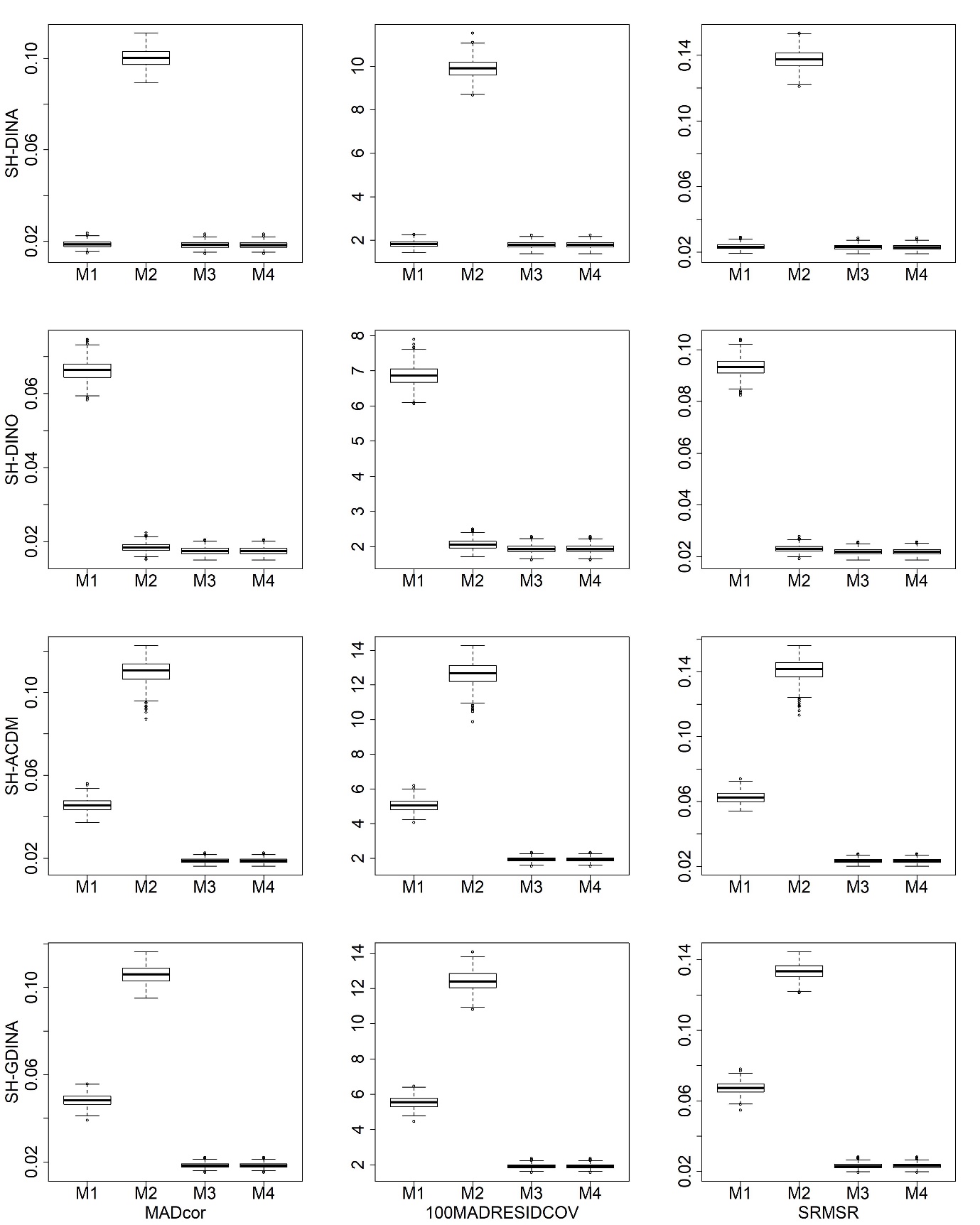


Figure S5. Boxplots of AFIs for *linear* attribute structures with

small sample size in high item quality cases


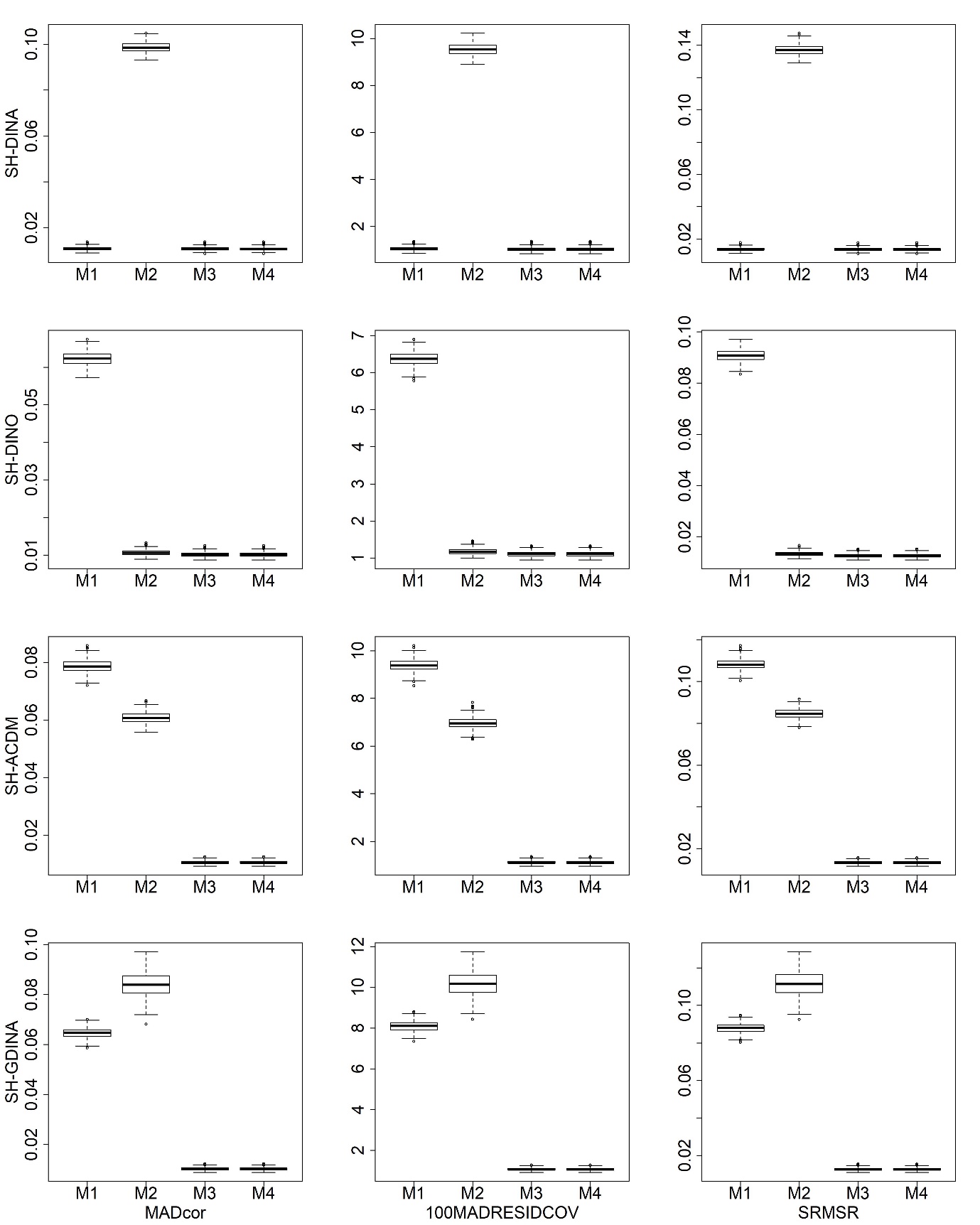


Figure S6. Boxplots of AFIs for *linear* attribute structures with

large sample size in high item quality cases


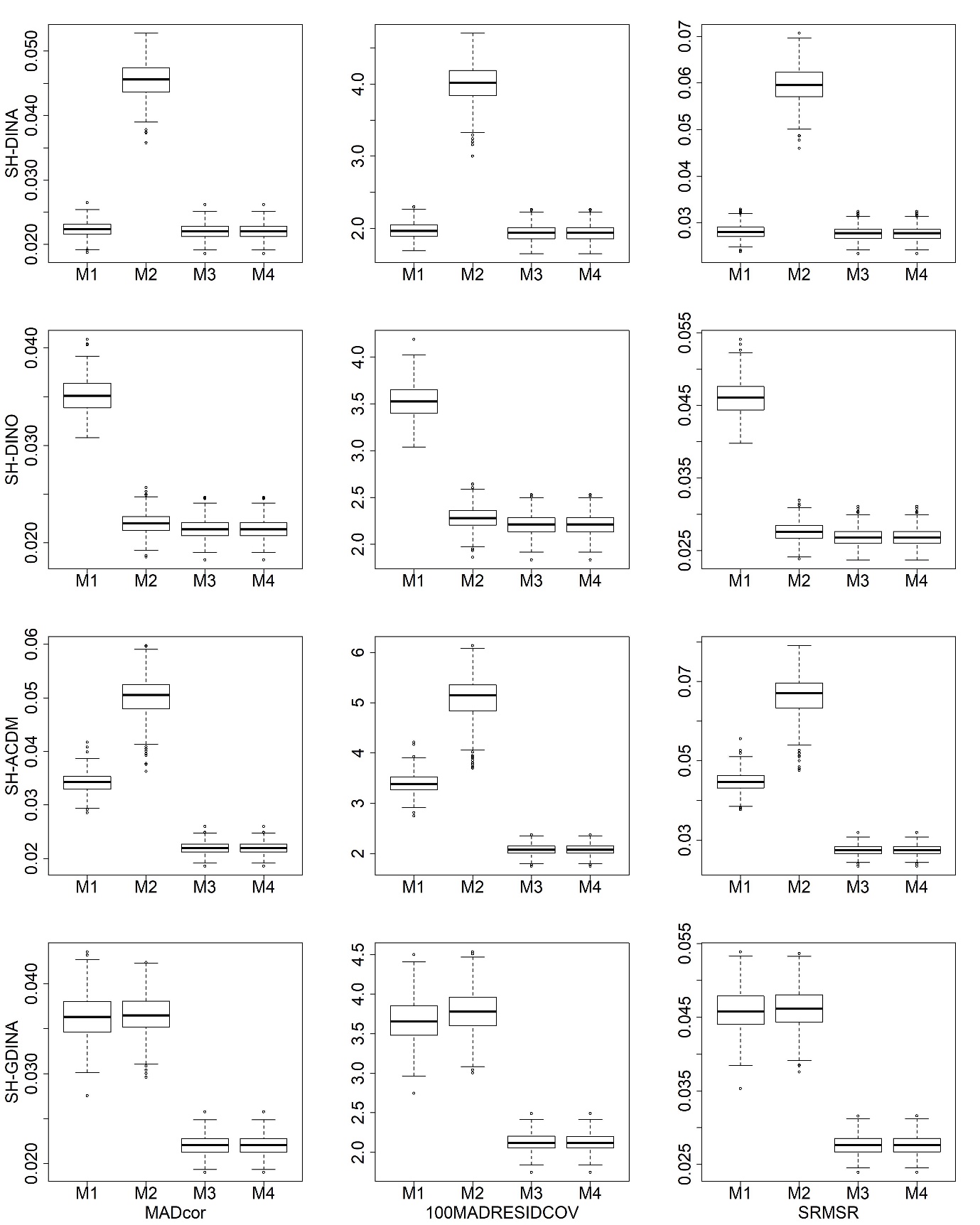


Figure S7. Boxplots of AFIs for *linear* attribute structures with

small sample size in low item quality cases


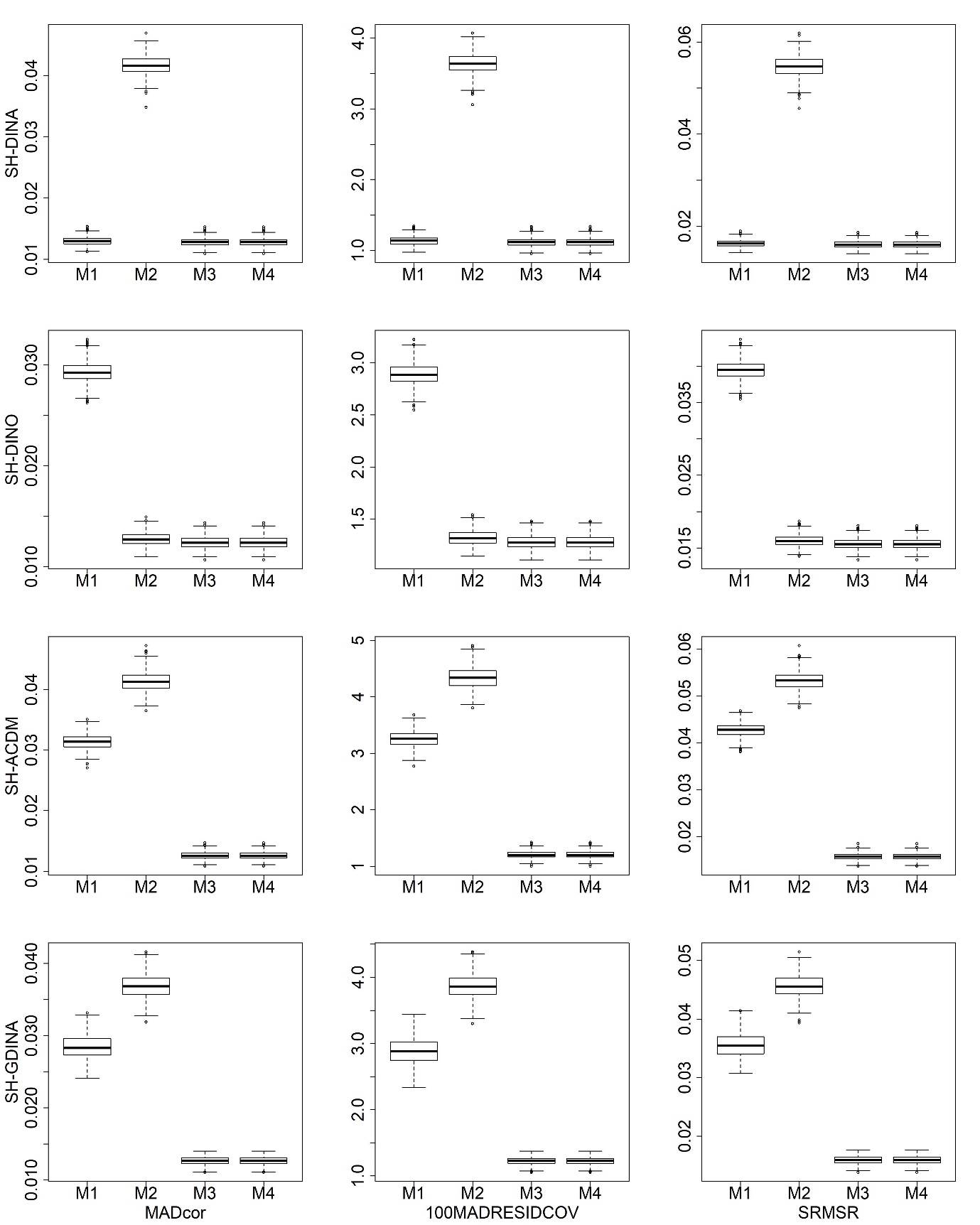


Figure S8. Boxplots of AFIs for *linear* attribute structures with

large sample size in low item quality cases


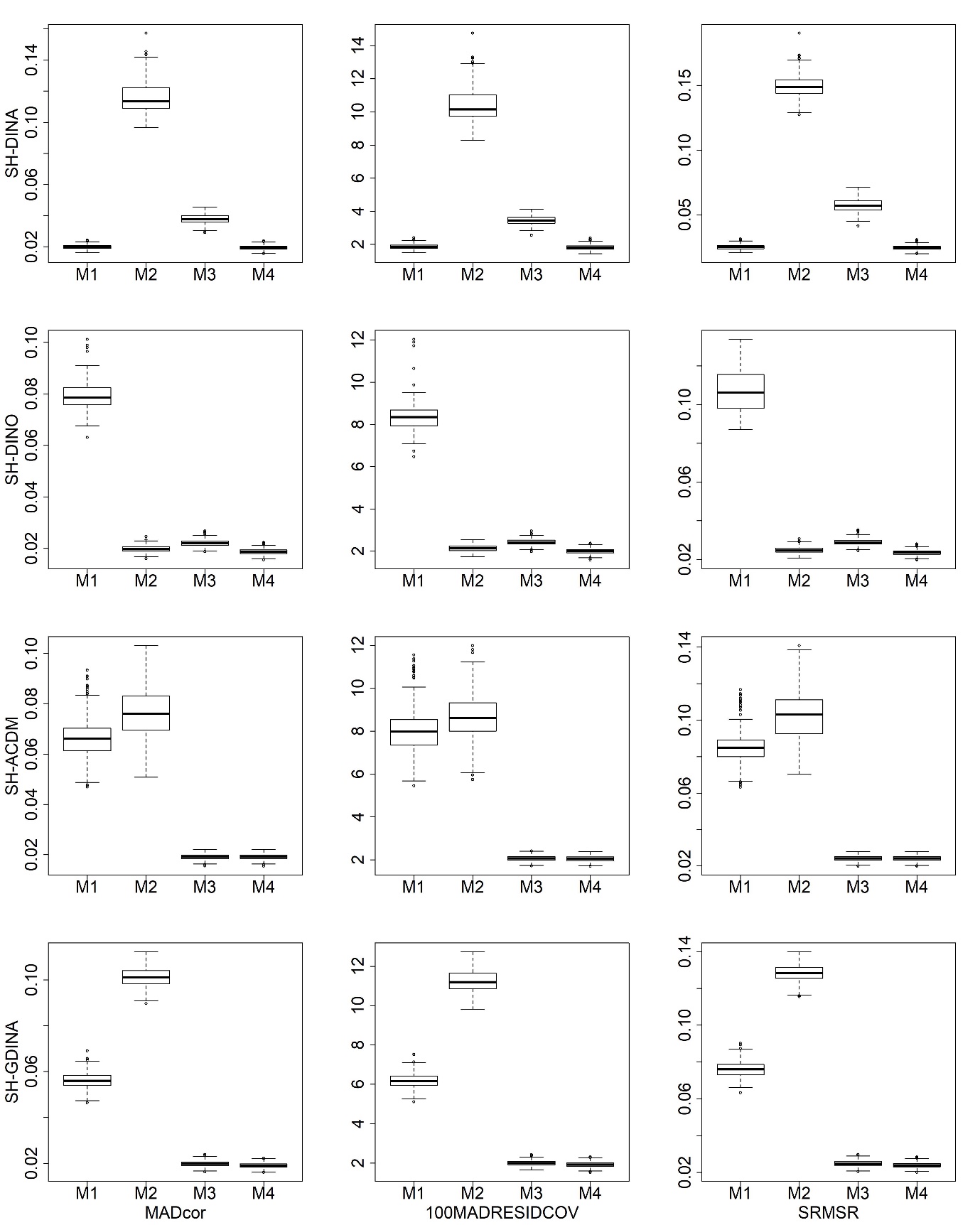


Figure S9. Boxplots of AFIs for *convergent* attribute structures

with small sample size in high item quality cases


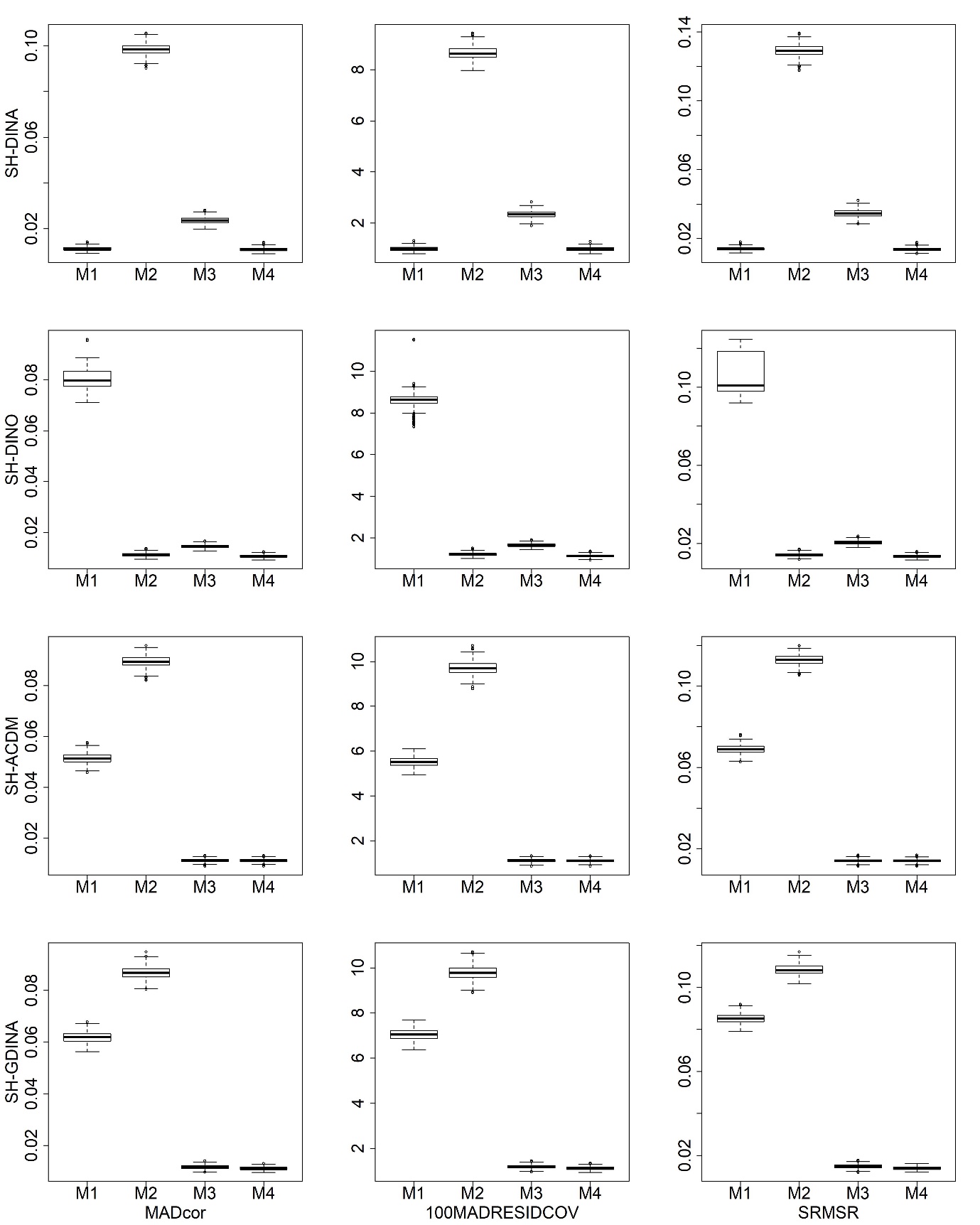


Figure S10. Boxplots of AFIs for *convergent* attribute structures

with large sample size in high item quality cases


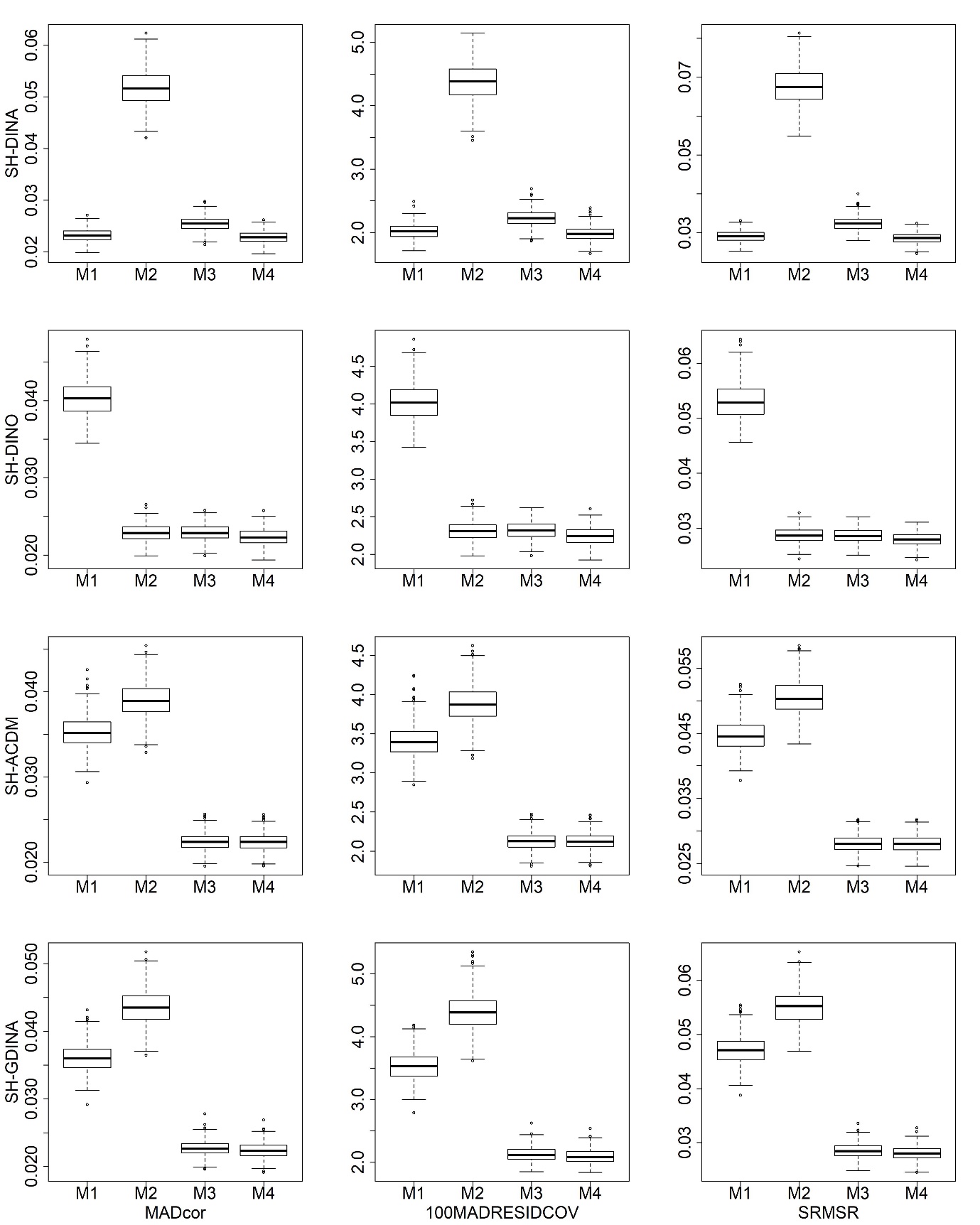


Figure S11. Boxplots of AFIs for *convergent* attribute structures

with small sample size in low item quality cases


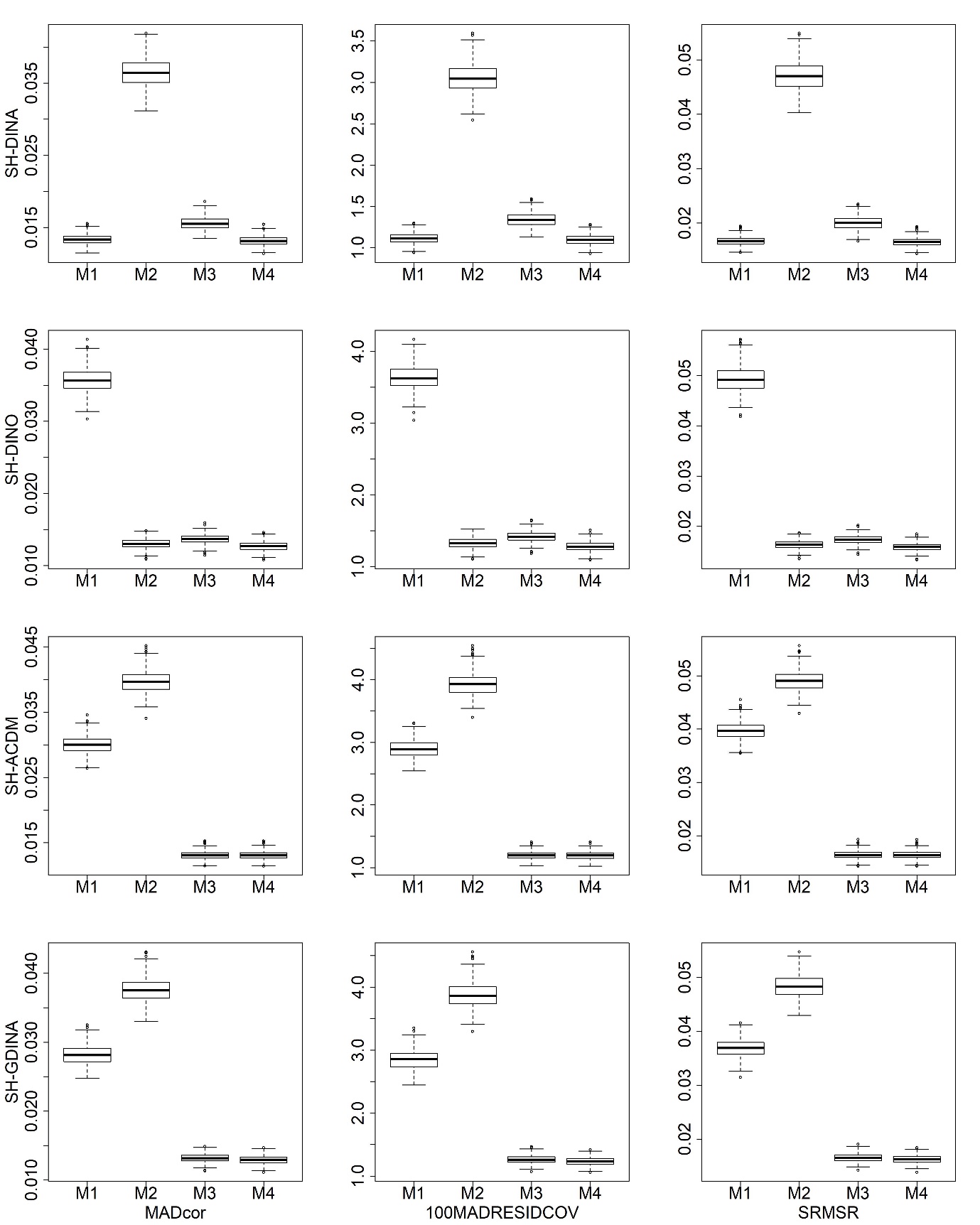


Figure S12. Boxplots of AFIs for *convergent* attribute structures

with large sample size in low item quality cases


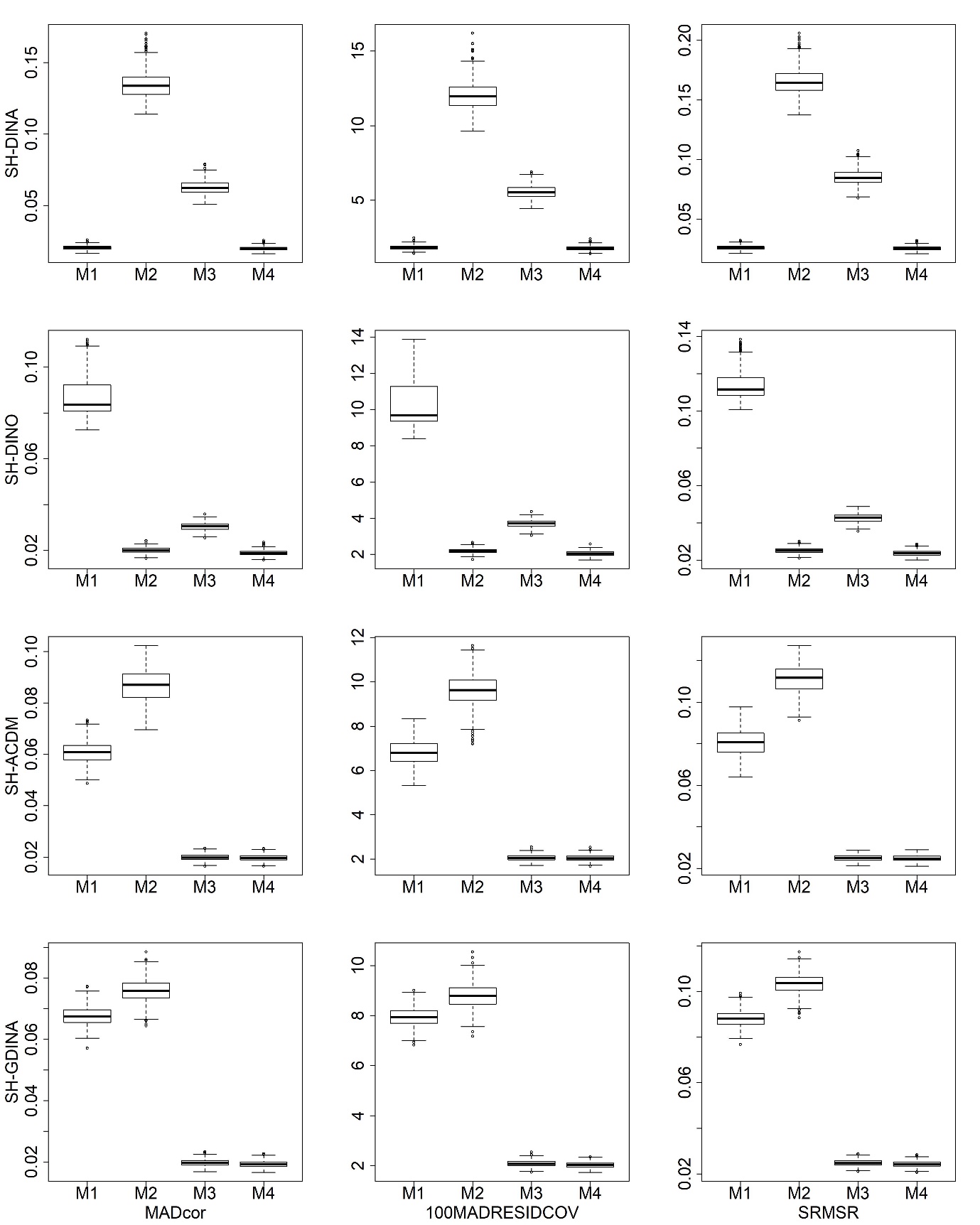


Figure S13. Boxplots of AFIs for *divergent* attribute structures

with small sample size in high item quality cases


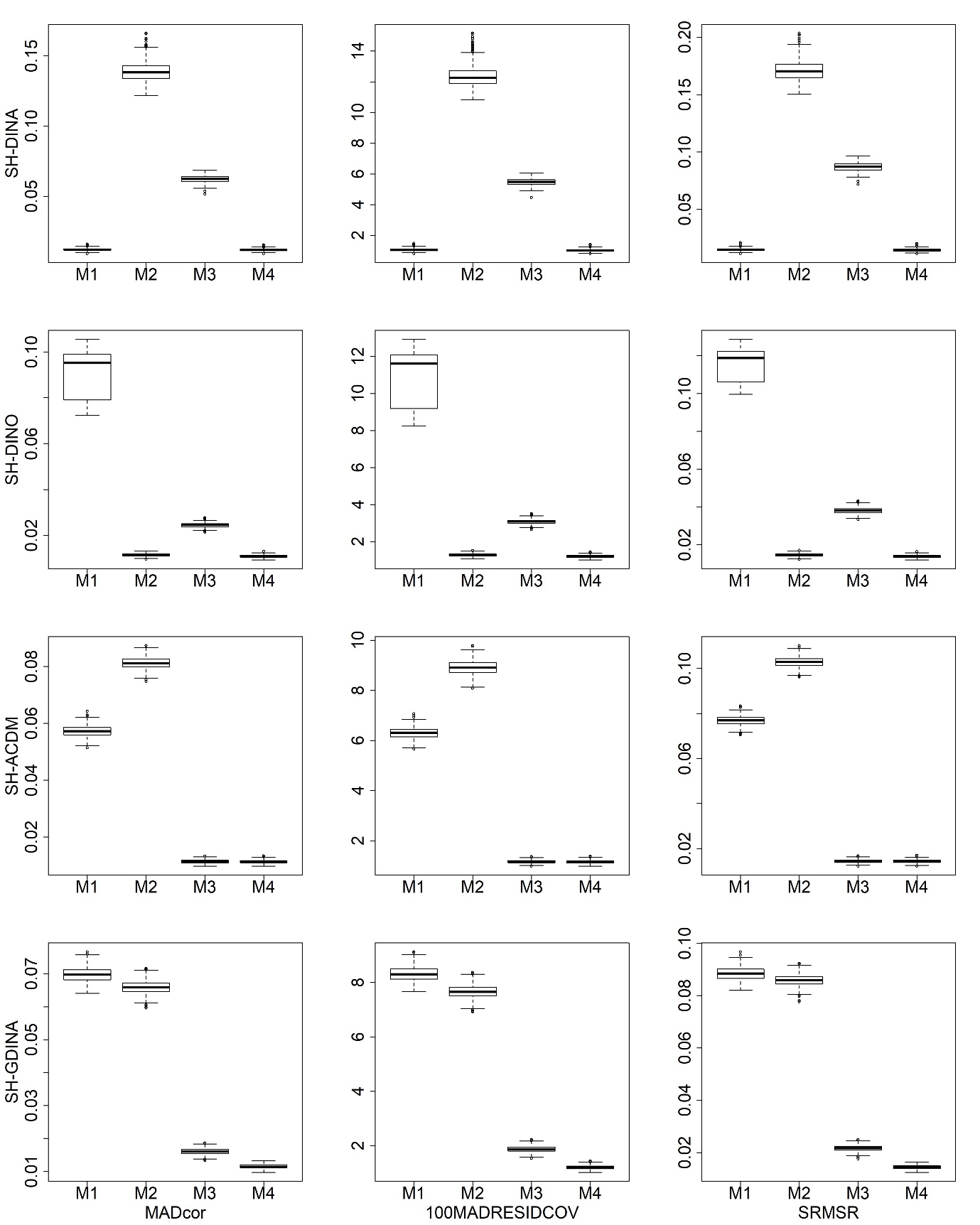


Figure S14. Boxplots of AFIs for *divergent* attribute structures

with large sample size in high item quality cases


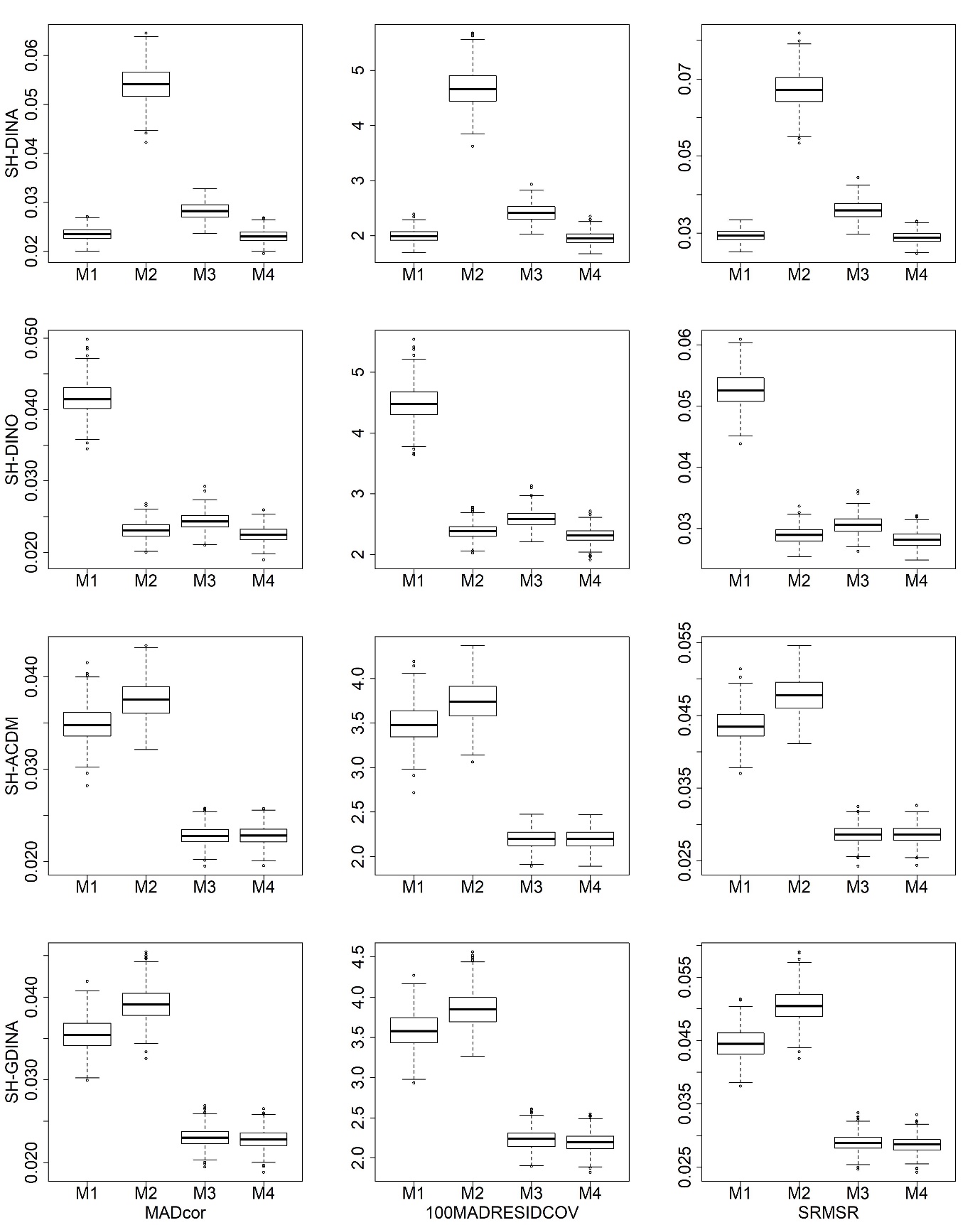


Figure S15. Boxplots of AFIs for *divergent* attribute structures

with small sample size in low item quality cases


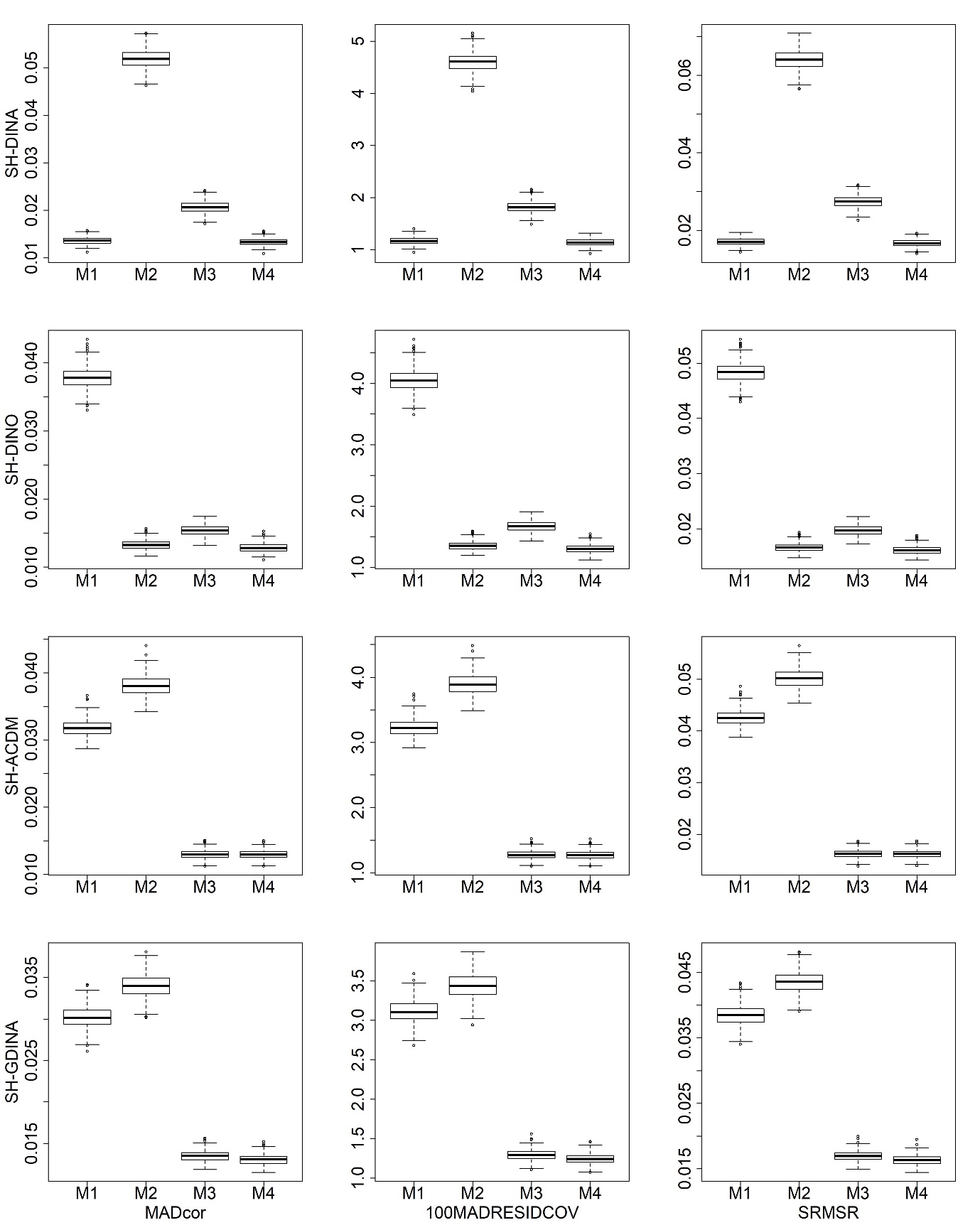


Figure S16. Boxplots of AFIs for *divergent* attribute structures

with large sample size in low item quality cases


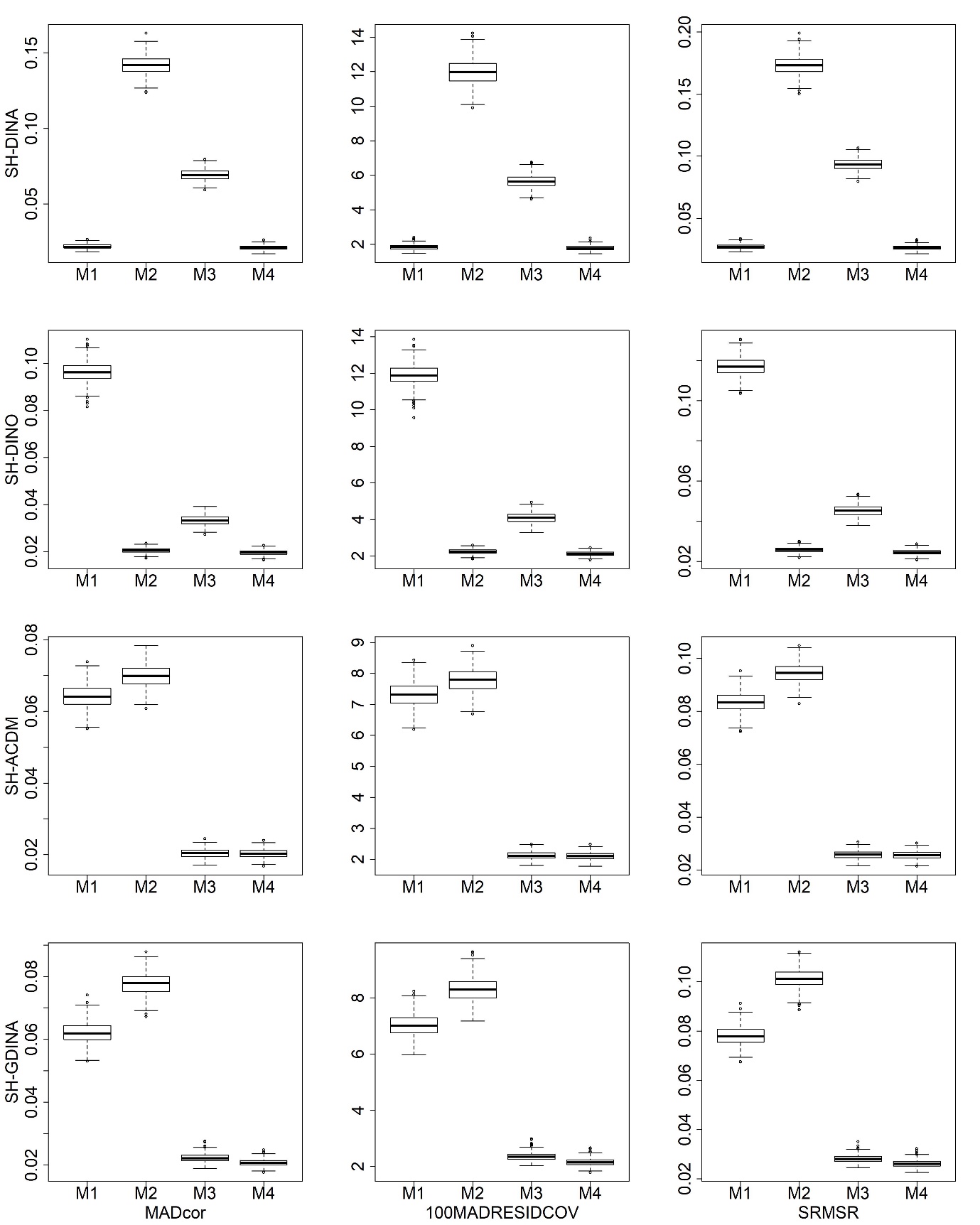


Figure S17. Boxplots of AFIs for *unstructured* attribute structures

with small sample size in high item quality cases


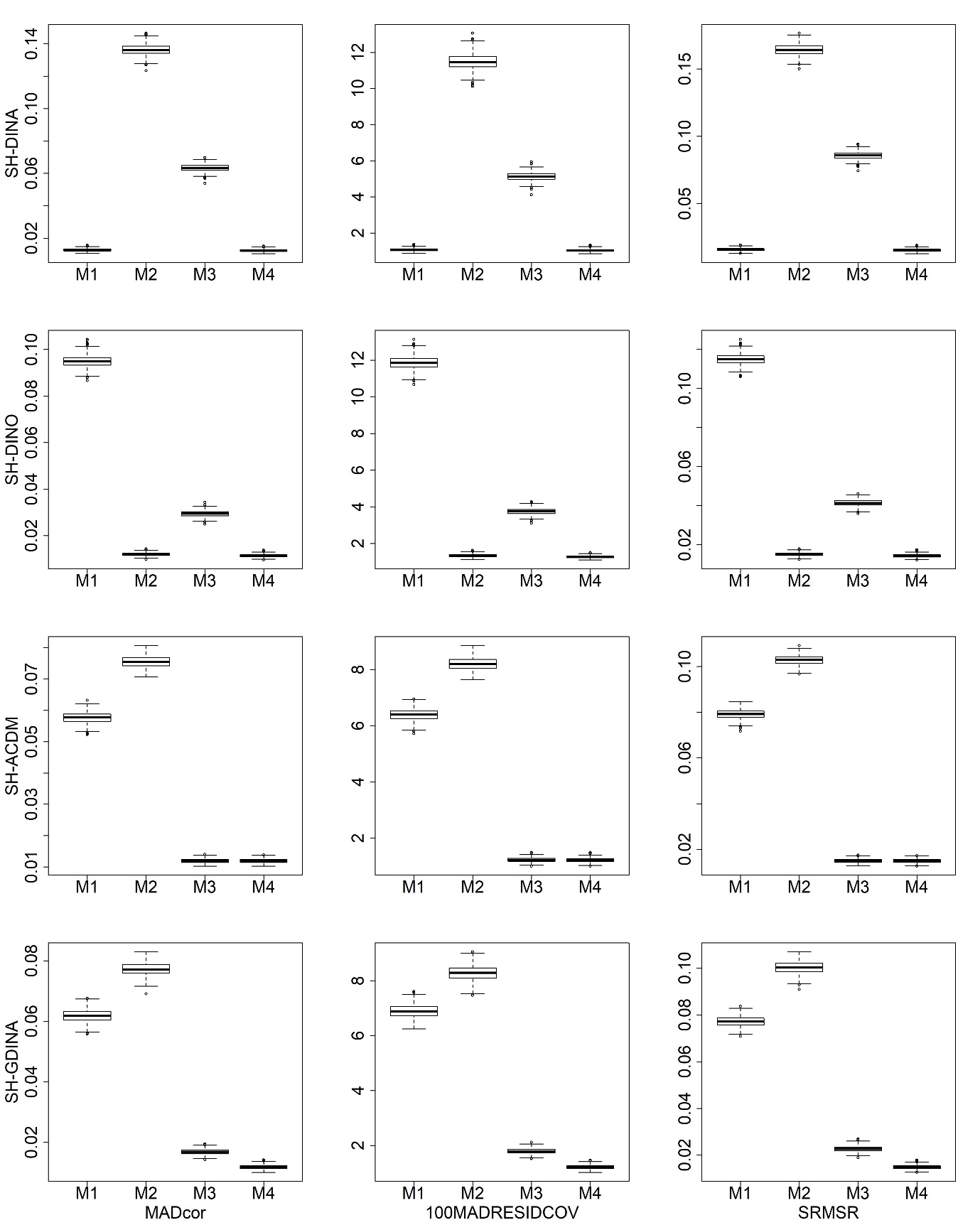


Figure S18. Boxplots of AFIs for *unstructured* attribute structures

with large sample size in high item quality cases


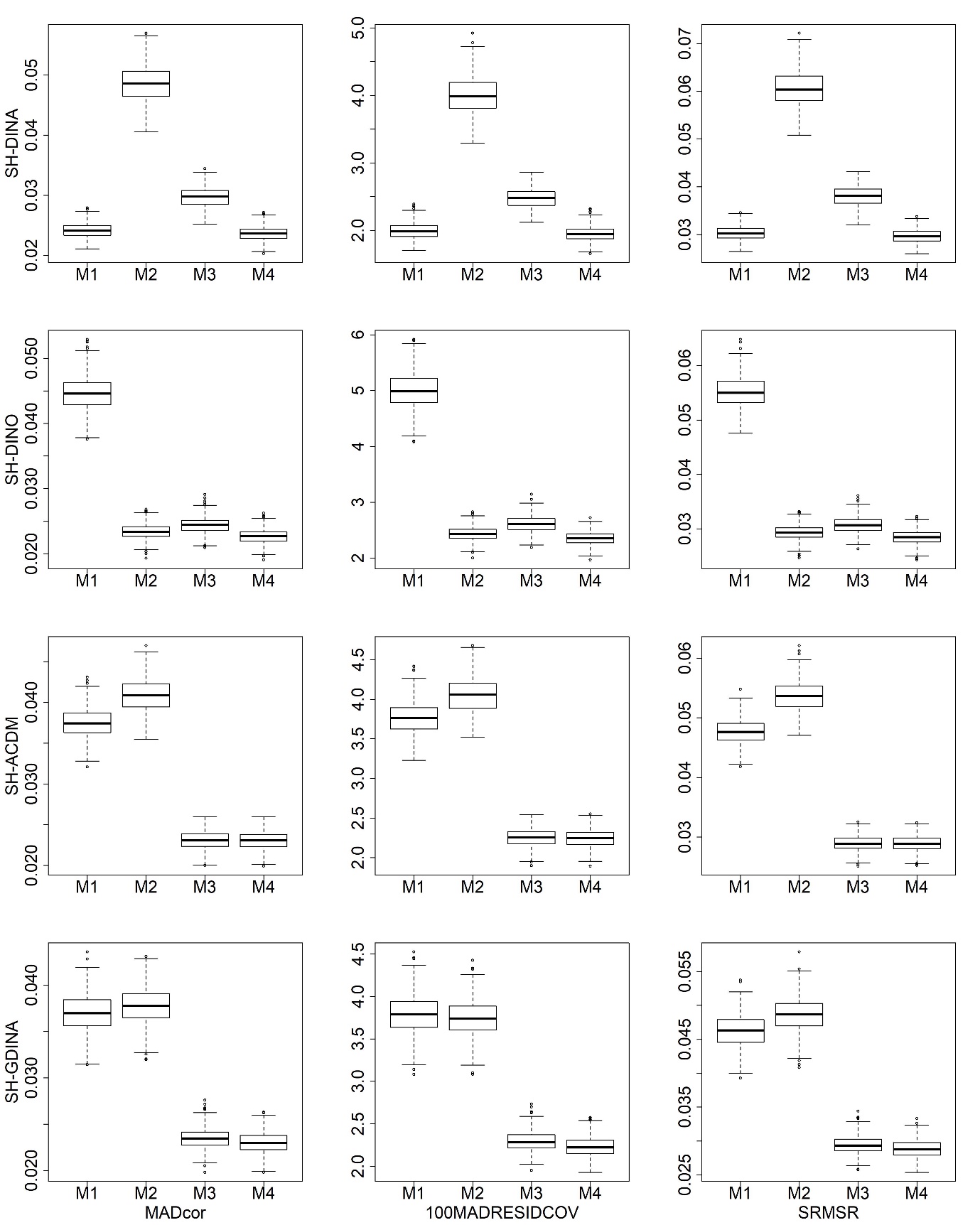


Figure S19. Boxplots of AFIs for *unstructured* attribute structures

with small sample size in low item quality cases


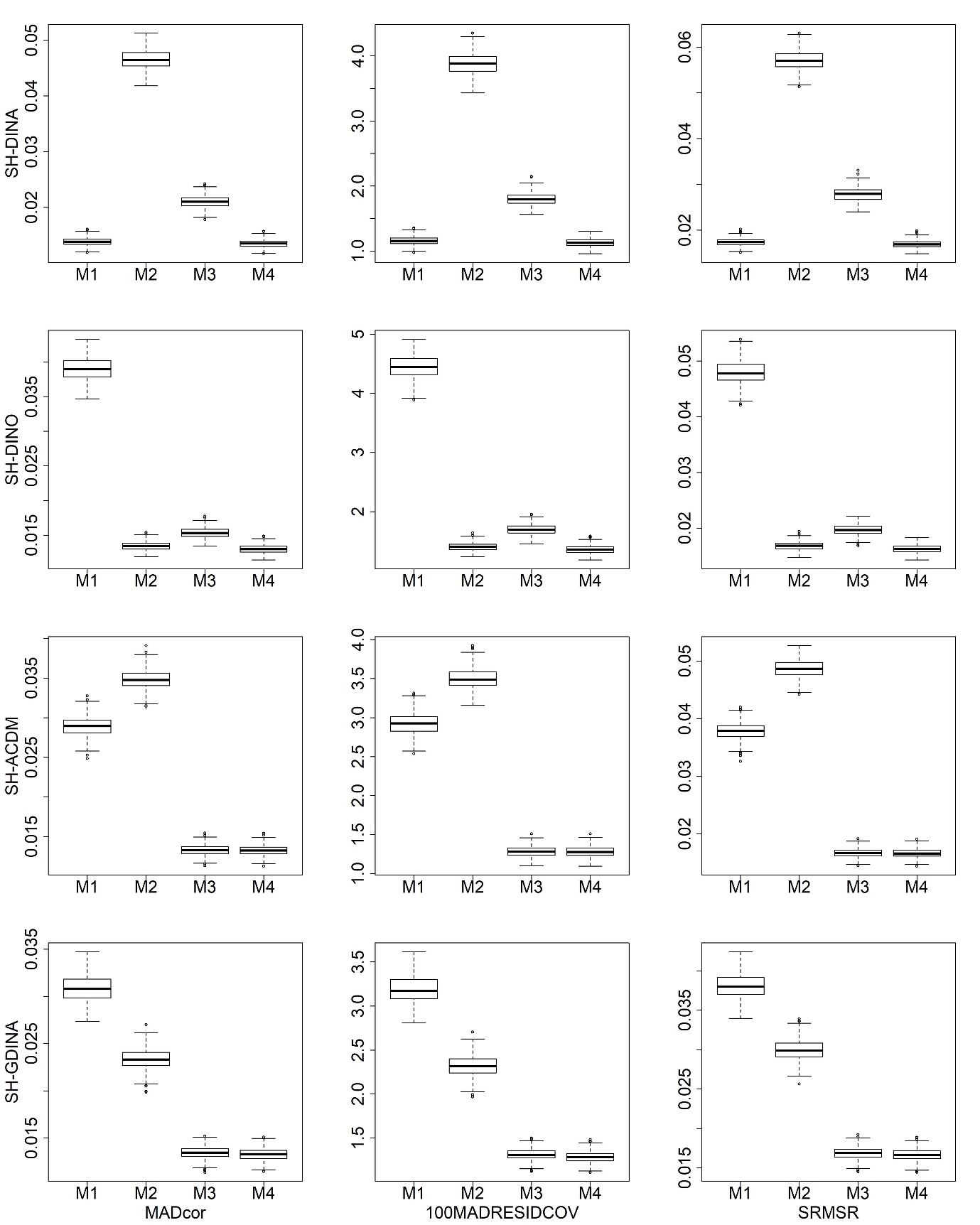


Figure S20. Boxplots of AFIs for *unstructured* attribute structures

with large sample size in low item quality cases
