# Supplementary material for: On the Sequential Hierarchical Cognitive Diagnostic Model
Source: Front Psychol. 2020 Oct 7;11:579018. doi: 10.3389/fpsyg.2020.579018 (PMC7577049; doi:10.3389/fpsyg.2020.579018)
Supplement: Supplementary file 2 [file Data_Sheet_2.docx]

**Appendix A. Source code for Simulation Study II**

rm(list=ls())

gc()

library(GDINA)

setwd("D:/code/CDM fit")

# This function is uded to calculate fit statistics for SH-CDMs.

# We refer to the R function "modelfit" in the GDINA package to define our function.

Fit_stat <- function (GDINA.obj, ItemOnly = FALSE)

{

## The codes are same as those in the GDINA package

## Upon the codes we obtain the key quantities for the fit statistics

if (extract(GDINA.obj, "ngroup") != 1) {

stop("modelfit is only applicable to single group analysis.",

call. = FALSE)

}

delta <- extract(GDINA.obj, "delta.parm")

Q <- extract(GDINA.obj, "Q")

if (max(Q) > 1) {

stop("modelfit is only available for dichotomous attribute models.",

call. = FALSE)

}

Qc <- extract(GDINA.obj, "Qc")

item.no <- c(Qc[, 1])

dat <- as.matrix(extract(GDINA.obj, "dat"))

N <- nrow(dat)

nitems <- ncol(dat)

ncat <- extract(GDINA.obj, "ncat")

K <- extract(GDINA.obj, "natt")

att <- as.matrix(extract(GDINA.obj, "attributepattern"))

L <- extract(GDINA.obj, "nLC")

Kj <- extract(GDINA.obj, "Kj")

nparJ <- npar(GDINA.obj)$`No. of total item parameters`

models <- extract(GDINA.obj, "models")

post <- c(extract(GDINA.obj, "posterior.prob"))

pj <- extract(GDINA.obj, "LCprob.parm")

pf <- extract(GDINA.obj, "LCpf.parm")

crossp <- GDINA:::crossprod.na(dat, dat, val = 0)/crossprod(!is.na(dat), !is.na(dat))

p <- c(colMeans(dat, na.rm = TRUE), crossp[lower.tri(crossp)])

Xi <- GDINA:::Mord(item.no, as.matrix(pj), post)

Xi2 <- cbind(rbind(Xi$Xi11, Xi$Xi21), rbind(t(Xi$Xi21), Xi$Xi22))

e <- c(Xi$uni, Xi$bi[lower.tri(Xi$bi)])

se <- sqrt(diag(Xi$bi) - c(Xi$uni)^2)

difr <- cor(dat, use = "pairwise.complete.obs") - (Xi$bi - Xi$uni %*% t(Xi$uni))/(se %*% t(se))

## Above this position, those are the same codes.

## fit statistics

logLik <- extract(GDINA.obj,"logLik")

Numpar <- nparJ+nrow(att)-1

SRMSR <- sqrt(sum((difr[lower.tri(difr)])^2/(nitems * (nitems - 1)/2)))

difdov <- cov(dat) - (Xi$bi - Xi$uni %*% t(Xi$uni))

MADRESIDCOV <- sum(abs(difdov[!lower.tri(difdov)]))/(nitems * (nitems+1)/2)

MADcor <- sum(abs(difr))/(nitems * (nitems-1))

AIC <- extract(GDINA.obj,"AIC")

AICc <- AIC + 2*Numpar*(Numpar+1)/(N-Numpar-1)

BIC <- extract(GDINA.obj,"BIC")

aBIC <- -2*logLik + Numpar*log((N+2)/24)

CAIC <- extract(GDINA.obj,"CAIC")

SABIC <- extract(GDINA.obj,"SABIC")

# Define outputs

output <- matrix(c(MADcor,100*MADRESIDCOV,SRMSR,AIC,BIC,aBIC,CAIC,AICc),nrow=1,

dimnames= list(c(),c("MADcor","100MADRESIDCOV","SRMSR","AIC","BIC","aBIC","CAIC","AICc")))

output

}

# We provide the SH-GDINA case with N=1000, high quality as an example.

N <- 1000 # sample size; or 3000

J <- 20 # number of polytomously scored items

K <- 4 # number of attributes

RC <- 3 # number of categories; or 4

# Generate candidates of Q-matrix's row-vectors

q <- as.list(1:K)

for(k in 1:K){

q[[k]] <- c(0,1)

}

Pat <- as.matrix(expand.grid(q))

## Attribute hierarchical stuctures

# non-hierarchical

struc1 <- list()

struc1$att.str <- Pat

struc1$att.prob <- rep(1/2^K,2^K)

struc1$str <- NULL

# linear structure A1->A2->A3->A4

linear <- list(c(1,2),

c(2,3),

c(3,4))

struc2 <- att.structure(linear,K)

struc2$str <- linear

# convergent structure A1->A2->A4

# A1->A3->A4

# A4 needs both A2 and A3

convergent <- list(c(1,2),

c(1,3),

c(2,4),

c(3,4))

struc3 <- att.structure(convergent,K)

struc3$str <- convergent

# divergent structure A1->A2->A3

# A2->A4

divergent <- list(c(1,2),

c(1,3),

c(3,4))

struc4 <- att.structure(divergent,K)

struc4$str <- divergent

# independent structure A1->A2;A1->A3;A1->A4

independent <- list(c(1,2),

c(1,3),

c(1,4))

struc5 <- att.structure(independent,K)

struc5$str <- independent

##########################

# Item quality level

Qua="High"# item quality; or "Low"

# Use number to denote attribute hierarchical stuctures

Hie=1 # attribute structure; 1 or 2, 3,4 5

Rep <- 500 # number of replications

# A file name to save .RData

filename <- paste0("TestHie_", as.character(Hie),"__Q_", Qua,".RData")

# Generate all possiable q-vectors,

Qs <- Pat[rowSums(Pat)==1|rowSums(Pat)==2|rowSums(Pat)==3,]

colnames(Qs) <- paste0("A",1:K)

# Generate identity-matrix

IK <- cbind(Item=c((J+1):(J+K)),Cat=rep(1,K),diag(K))

# Generate Q-matrix

temp_Q <- Qs[sample(1:nrow(Qs),J*RC,replace=T),]

temp_Q <- cbind(Item=rep(1:J,each=RC),Cat=rep(1:RC), temp_Q) #Q-completion (add some Item number and C)

Q <- rbind(temp_Q,IK)

# True model

T_models <- c(rep("GDINA",nrow(Q)))

# save the parameter delta

M <- array(dim=c(5,8,Rep),dimnames= list(c(),c("MADcor","100MADRESIDCOV","SRMSR","AIC","BIC","aBIC","CAIC","AICc"),c()))

for (i in 1:Rep){

# Generate response data

if (Qua=="High")

gs <- data.frame(guess=runif(nrow(Q),0.1,0.2),slip=runif(nrow(Q),0.1,0.2))

if (Qua=="Low")

gs <- data.frame(guess=runif(nrow(Q),0.2,0.3),slip=runif(nrow(Q),0.2,0.3))

struc <- switch(Hie, {struc1},{struc2},{struc3},{struc4},{struc5})

true.lc <- sample(c(1:2^K),N,replace=TRUE,prob=struc$att.prob)

true.att <- attributepattern(K)[true.lc,]

# simulate sequential GDINA model

simseq <- simGDINA(N, Q, sequential = TRUE, gs.parm = gs, model = T_models,attribute = true.att, att.dist ="saturated")

# simulated data

Y <- extract(simseq,what = "dat")

mod1 <- GDINA(Y, Q, model= T_models, sequential=T,att.str= struc1$str,att.dist ="saturated")

M[1,,i] <- (Fit_stat(mod1))

mod2 <- GDINA(Y, Q, model= T_models, sequential=T,att.str= struc2$str,att.dist ="saturated")

M[2,,i] <- (Fit_stat(mod2))

mod3 <- GDINA(Y, Q, model= T_models, sequential=T,att.str= struc3$str,att.dist ="saturated")

M[3,,i] <- (Fit_stat(mod3))

mod4 <- GDINA(Y, Q, model= T_models, sequential=T,att.str= struc4$str,att.dist ="saturated")

M[4,,i] <- (Fit_stat(mod4))

mod5 <- GDINA(Y, Q, model= T_models, sequential=T,att.str= struc5$str,att.dist ="saturated")

M[5,,i] <- (Fit_stat(mod5))

}

temp_rates <- apply(M,c(2,3),function(x){ x[is.na(x)] <- 1000; ind <- which.min(x); y <- rep(0,length(x)); y[ind] <-1; y })

select_rates <- apply(temp_rates,c(1,2),mean)

rownames(select_rates) <- c("non_hierarchical","linear","convergent","divergent","independent")

save(M,select_rates,file= filename)
